# Mesostructured Water Enhances Stability of ProteinMPNN-Designed Ubiquitin-Fold Proteins

**DOI:** 10.1021/jacs.5c19875

**Published:** 2026-02-05

**Authors:** Lu-Yi Chen, Wei-Lin Lu, Tanvi Pathania, I-Hsuan Chu, Meng-Ru Ho, Wei-Chen Chuang, Yuan-Chao Lou, Ta I. Hung, Yohei Miyanoiri, Chia-en A. Chang, Kuen-Phon Wu

**Affiliations:** † 38017 Institute of Biological Chemistry, Academia Sinica, Taipei 115201, Taiwan; ‡ Institute of Biochemical Sciences, College of Life Science, National Taiwan University, Taipei 106319, Taiwan; § Department of Chemistry, 8790 University of California Riverside, Riverside, California 92521, United States; ∥ Biomedical Translation Research Center, Academia Sinica, Taipei 115201, Taiwan; ⊥ Research Center for Next-Generation Protein Sciences, Institute for Protein Research, 13013 Osaka University, 3-2 Yamadaoka, Suita, Osaka 565-0871, Japan

## Abstract

AI-designed protein variants have demonstrated remarkable resistance to heat and chemical stress, yet the molecular mechanisms underlying this stability remain unclear. Here, we present a comprehensive biophysical and nuclear magnetic resonance (NMR) analysis of thermally stable ubiquitin and its ProteinMPNN-designed variants, R4 and R10, together with a second system based on the less stable ISG15 C-terminal domain (ISG15-CTD). Both R4/R10 and ProteinMPNN-designed ISG15-CTD variants (ICVs) exhibit extraordinary thermostability beyond 120 °C, and resist extreme denaturation at pH 3.0 in 8 M urea. NMR relaxation and hydrogen–deuterium exchange, and molecular-dynamics simulations reveal a protective mesostructured hydration shell that strengthens the hydrogen bonding network between protein-bound and bulk water, thereby suppressing unfolding. Sequence and electrostatic analyses indicate that this hydration arises from charge enrichment and clustering on the protein surface. These findings identify mesostructured hydration as a general, sequence-encoded mechanism of ProteinMPNN-driven stability and provide a physical framework for designing highly resilient biomolecules.

## Graphical abstract

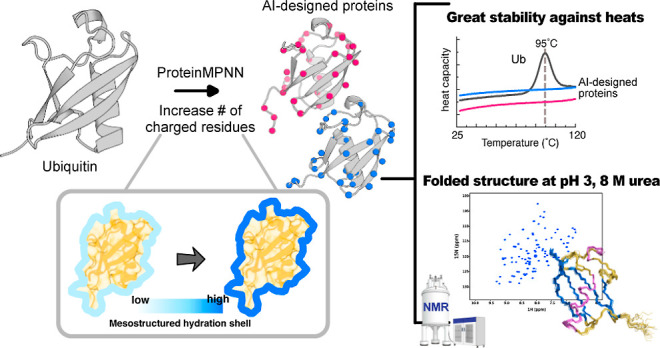

## Introduction

Protein folding and stability have been the subjects of intense research for decades,^1–3^ as scientists strive to understand the intricate interplay between a protein’s structure, dynamics, and function. The ability to design stable proteins has gained increasing importance across diverse fields, including biopharmaceuticals and biocatalysis, where robust proteins are essential. Traditional protein engineering strategies often focus on optimizing hydrophobic cores and reinforcing canonical interactions such as hydrogen bonds (HBs) and salt bridges to enhance stability.^4–6^ These strategies have been instrumental in improving protein function and folding efficiency, but they often require iterative experimental validation and are limited by the difficulty of modeling highly complex interactions. Over the past two decades, computational tools such as Rosetta,^7^ FoldX,^8^ FireProt,^9^ and Proteus^10^ have enabled physics- and knowledge-based prediction^11–13^ of stabilizing mutations through detailed modeling of hydrogen bonding, van der Waals packing, and electrostatics. These methods have achieved substantial success in improving thermostability and catalytic robustness while providing mechanistic interpretability of sequence–structure relationships. However, because they rely heavily on human intuition and rational design principles, they typically focus on modifying a limited number of residues at a time; the enormous complexity of protein sequence space still constrains systematic exploration of alternative stabilizing features beyond those observed in natural proteins.

To overcome these limitations, deployment of artificial intelligence (AI) has revolutionized the field of protein design by offering data-driven, computationally guided solutions that capture a more comprehensive understanding of protein folding, stability, and interactions.^14,15^ AI models leverage vast structural and sequence data sets to recognize the patterns governing protein behavior, enabling novel variants with enhanced properties to be designed. Unlike conventional methods, AI-driven approaches can encompass complex, nonintuitive interactions that influence stability and function, significantly broadening the scope of protein engineering.^16–19^ These advancements have facilitated the design of proteins displaying enhanced stability, solubility, and even novel functionalities that may not be accessible through traditional engineering techniques. Furthermore, reference-based methods, such as ProteinMPNN, offer a complementary approach, leveraging existing structural templates to computationally resequence proteins while preserving their three-dimensional architecture.^20^ ProteinMPNN extend these capabilities by learning from large structure–sequence data sets to explore a broader mutational landscape, complementing rather than replacing the established physics-based frameworks. Notably, ProteinMPNN has successful produced variants of ubiquitin, TEV protease, myoglobin, and de novo proteins that not only maintain their structural integrity, but also exhibit enhanced thermal stability.^20–22^

Ubiquitin (Ub), a highly conserved 76-residue protein, serves as an ideal model for engineering studies due to its exceptional stability and important biological roles.^23–25^ With a melting temperature (*T*_m_) of 95 °C and resilience under acidic conditions, Ub is a challenging subject for enhanced protein stability. Here, we present our discovery of ProteinMPNN-designed ubiquitin variants (UbVs) that incorporate mesostructured water, significantly improving their stability under extreme conditions. Designed initially for allosteric activation of the Rsp5 HECT E3 ligase, the ProteinMPNN-designed variants R4 and R10 exhibit increased melting temperatures and enhanced solubility.^21^ However, the molecular basis underlying their stability remained unclear. By using biophysical spectroscopy including NMR, circular dichroism (CD), and differential scanning calorimetry (DSC), as well as molecular dynamics simulations, we reveal that these ProteinMPNN-designed variants integrate mesostructured water molecules, conferring resistance to thermal and chemical denaturation. Our findings provide valuable insights into the mechanisms by which deep learning design strategies can enhance protein stability, expanding the potential for engineering robust biomolecules. The incorporation of mesostructured water, which forms an intricate network of HBs and structured water molecules surrounding a protein, appears to be a key factor in the enhanced stability of both these ProteinMPNN-designed Ub variants. This hydration shell potentially acts as a protective shield, insulating the protein from thermal and chemical denaturation and contributing to the remarkable heat resistance and acid resilience observed in these engineered proteins. These insights into the role of mesostructured water in protein stability open up new avenues for the design of even more robust and stable biomolecules using future AI-based techniques.

## Results

### Thermally Stable ProteinMPNN-Designed Ubiquitin Variants

Ubiquitin (Ub) is a folded β-grasp protein consisting of five β-strands and two α-helices and it has several biological paralogs, including NEDD8, SUMO, and ISG15, with the *ISG15* gene encoding two Ub-fold domains (Figure S1a).^23,26^ We wondered if the structurally homologous Ub-fold and engineered Ub proteins share similar conformational stabilities. We employed DSC to measure the thermal profiles of selected Ub-fold proteins, revealing a significant reduction in stability between Ub (*T*_m_ 96.8 °C) and ISG15 77–157 (62.5 °C). The *T*_m_ of Ub also declined to 76.4 °C when it transitioned from a neutral to acidic environment (pH 3.0). Additionally, the Ub variant (UbV) R5.4,^27^ screened by phage display and exhibiting five and two residual replacements and additions, respectively, presented a 10 °C reduction in *T*_m_ (85.1̊C) (Figure 1a). This reduced thermal stability of R5.4 is similar to our findings reported previously on the phage-displayed UbVs ME.2 and ME.4, both of which were raised against MERS-CoV papain-like protease.^5^

**Figure 1:**
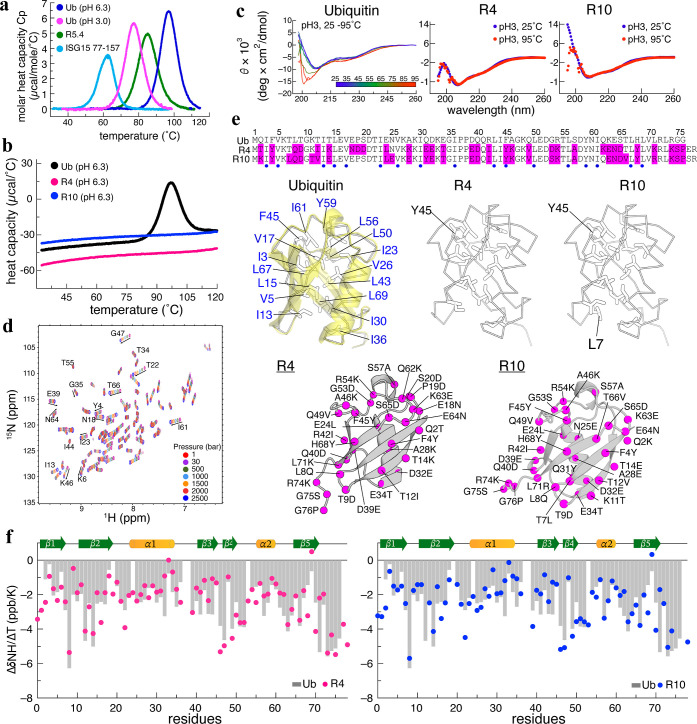
Thermophilic and structural profiles of UbVs R4 and R10 (a). The melting temperatures of ubiquitin (Ub) at pH 6.3 and pH 3.0, the ubiquitin-fold domain of ISG15 (residues 77–157), and the phage display-selected UbV R5.4. Ub at pH 6.3 remains stable up to 96 °C. (b) The minimal changes in heat capacity of R4 and R10 across different temperatures evidence their exceptional stability. (c) Far-UV CD spectra of R4 and R10 at pH 3.0 reveal similar secondary structural distributions. Heating R4 and R10 up to 95 °C resulted in identical CD profiles, indicating their thermal stability. In contrast, Ub at pH 3.0 undergoes unfolding from 25 to 95 °C, displaying a characteristic random-coil minimum near 198 nm. (d) Pressure-dependent NMR HSQC spectra of R4 recorded from 1 to 2500 bar indicate that R4 maintains a well-folded conformation even at 2500 bar. (e) Aligned sequences of Ub, R4, and R10 with indicated conserved hydrophobic residues (blue dot) and substitutions (pink highlights). In the structural representation, hydrophobic residues in Ub are marked in stick mode, with 13 core residues labeled in blue. R4 and R10 retain 12 of these core residues, except for an F45Y-substitution. Additionally, R10 features an extra hydrophobic residue, L7, located near the core. Substitutions between Ub and its variants are mapped onto the R4 and R10 structures where most changes occur along the β-strand surface. (f). NMR temperature coefficients (Δδ_NH_/Δ*T*) of Ub, R4, and R10 are represented by gray bars, red dots, and blue dots, respectively. The correlation plots of Δδ_NH_/Δ*T* are presented in Supporting Information Figure S5e.

Next, we investigated the DSC profiles of the R4 and R10 UbVs designed by ProteinMPNN, an inverse-folding tool that was used to redesign the Ub primary sequence based on the 3D structure of Rsp5-Ub.^21^ Surprisingly, we observed that R4 and R10, which host 33 and 32 substitutions relative to wild-type Ub, respectively, exhibit remarkable heat resistance. The heat capacity curves remained largely unchanged, indicating that the *T*_m_ for R4 and R10 are well above 120 °C (Figure 1b). It is not trivial to rapidly and significantly improve thermal stability from such a small, compact, and highly stable protein such as Ub by means of one-shot modification, in particular for one in which 43% residues had been changed. This enhanced thermal stability seems to be common to our ProteinMPNN-designed UbVs, as six additional ubiquitin variants also presented superior *T*_m_ values (Figure S1c). However, it is unclear how ProteinMPNN had achieved the elevated thermal resistance and which residues are involved.

CD and DSC measurements further uncovered that R4 and R10 are promisingly heat resistant up to 95 °C under acidic pH 3.0 conditions (Figure 1c). In contrast, Ub exhibits the characteristic random-coil minimum at 198 nm in the CD spectrum at pH 3.0 and 95 °C, exceeding its *T*_m_ (76 °C). The stability of R4 and R10, compared to Ub, substantially improved when the pH shifted from 6.3 to 3.0. Notably, R4 and R10 never unfolded during the DSC and CD characterizations and they proved stable for more than 24 months in the NMR tubes. Next, we examined the folding stability of R4 under varied hydrostatic pressures, ranging from 1 to 2500 bar, by means of NMR spectroscopy. The high-pressure NMR ^15^N-HSQC spectra of R4, like that of Ub reported previously,^28^ remained well-dispersed at 2500 bar, indicating that R4 retains a compact and folded conformation under extreme pressure (Figure 1d).

We noted that the UbVs R4 and R10 exhibited remarkable stability relative to native Ub under various conditions, including heat, acid, and high pressure, though the exact mechanisms were not entirely clear. We reasoned that the AI tool ProteinMPNN had modified the hydrophobic core or HB network of these variants, thereby effecting their enhanced stability. Structural analyses revealed that the crystal structures of R4 (PDB 9LQM, this study) and R10 (PDB 9LQK, this study) at resolutions of 1.4 and 1.5 Å (Table S1), respectively, are profoundly similar to that of Ub, i.e., within C^α^ RMSD of 0.4 Å. Thirteen residues constitute the hydrophobic core of Ub, and 12 of those were unchanged in R4 and R10 (Figure 1e). The single substitution, i.e., F45 to Y45 in both R4 and R10, is unlikely to dramatically impact protein stability, as the polarity and hydrophobicity of other core residues, such as V17, I36, L50, L56, I61, and Y59, remained largely preserved.

Since the hydrophobic cores of R4, R10, and Ub presented only subtle differences, next we investigated the intramolecular HB networks and bond strengths using the corresponding crystal structures and NMR temperature coefficients Δδ_NH_/Δ*T*, respectively. Our analysis of the crystal structures of R4, R10, and Ub revealed that the intramolecular backbone of the HB network in Ub is preserved in both R4 and R10, though R4 and R10 have four additional short-range HB (Figure S2). NMR Δδ_NH_/Δ*T* values revealed that the temperature dependency of amides in R4 and R10 is largely consistent with that of Ub as the correlation coefficients of 0.94, implying identical HB strengths and networks between these proteins^29–31^ (Figures 1f and S3, S4, S5). There is an empirical rule that a Δδ_NH_/Δ*T* value greater than −5.0 or −2.72 ppb/K for Ub^31^ or protein GB3,^30^ respectively, is associated with HB. For example, in Ub, R4, and R10, the L69 amide at β5, which forms a HB with the distant K6 carboxyl (β1) to maintain intersheet interactions, exhibits similar NMR Δδ_NH_/Δ*T* values of −0.64, 0.49, and 0.33 ppb/K, respectively, indicative of a strong hydrogen-bonded amide. K71 of R4 and R71 of R10 exhibit pronounced changes, with their Δδ_NH_/Δ*T* values elevated by approximately 3.1 ppb/K, whereas L71 of Ub displays a markedly lower value of −5.3 ppb/K. Notably, the amide groups of K71 and R71 engage in hydrogen bonds with solvent molecules in the crystal structures, providing a plausible explanation for the elevated temperature coefficients. Combining the visualized HB networks in crystal structures and NMR Δδ_NH_/Δ*T* profiles, both HB strength and the HB network are well preserved in the engineered variants, contributing to their stability under various conditions, though how this enhanced resistance to challenging conditions is achieved remains unknown. Interestingly, there are notable differences among the three proteins in terms of NMR relaxation parameters, including ^15^N–R_1_ and ^15^N–R_2_. Specifically, the ProteinMPNN-designed variants R4 and R10 display strikingly increased and reduced ^15^N–R_2_ and ^15^N–R_1_ values, respectively, compared to Ub (Figure 2a). R10 might undergo conformational fluctuations at several residues at the μs-ms time scale, as suggested by elevated R2/R1 ratios at residues T11, I13, K48, and the C-terminus relative to the baseline. By comparison, R4 lacks such regional increases, consistent with a more rigid behavior appearing fast (ns-ps) motions. Moreover, the ^15^N–R_2_ rates of R4 and R10 are on average 2–4 Hz higher than those of wild-type Ub. Our previous analyses confirmed that R4, R10, and Ub are monomeric in solution,^21^ as corroborated by NMR diffusion-ordered spectroscopy (DOSY) measurements, which showed minimal differences in their translational diffusion coefficients *D*_trans_ (Figure S6a). Consequently, the increased ^15^N–R_2_ rates of R4 and R10 are not attributable to protein oligomerization. Despite the indistinguishable three-dimensional structures and similar HB profiles, the enhanced thermal and acidic stability of R4 and R10 over Ub, as well as their elevated ^15^N–R_2_ dynamics, imply that differences might be linked to the hydrophilic dynamics, such as their protein-water interactions, may underlie their improved stability.

**Figure 2:**
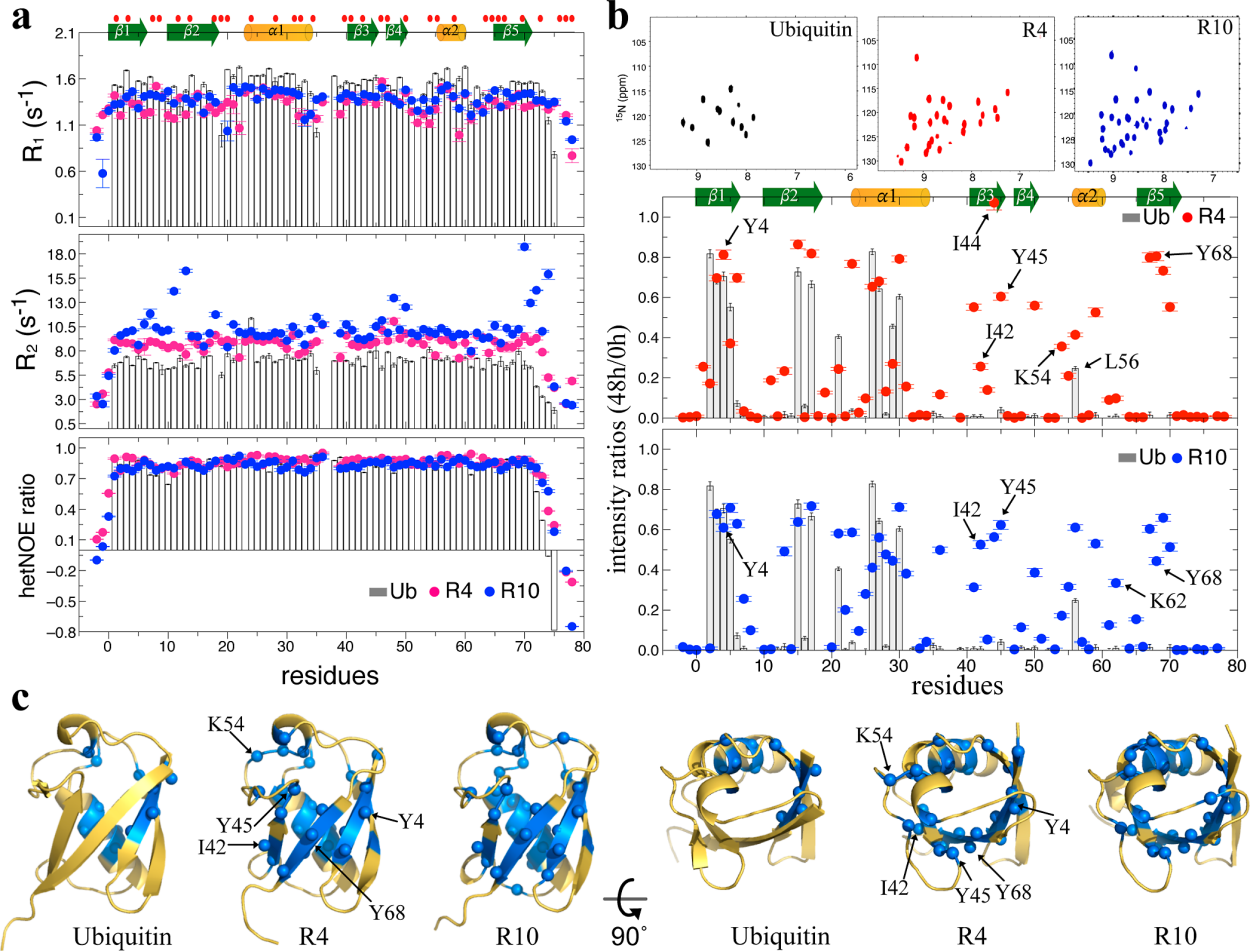
UbVs may possess a different hydration shell to that of native Ub. (a). The ^15^N R_1_, R_2_, and heteronuclear NOE values for Ub, R4, and R10 are represented by gray bars, red dots, and blue dots, respectively. R4 and R10 exhibit ^15^N–R_2_ rates that are 2–5 Hz higher than those of Ub. (b). NMR spectra of Ub, R4, and R10 48 h after exchange into 100% D_2_O reveal that R4 and R10 retain more unchanged residues than Ub. The intensity ratios of spectral cross-peaks at 48 and 0 h for each protein are also presented. (c). Residues retaining ≥20% of their original signal intensity (ratio ≥0.2) are highlighted in blue and are represented as spheres in the corresponding structures. The hydrophobic I44 patch in R4 and R10 retains a significant number of unchanged amide signals, whereas these signals are fully exchanged in Ub.

### The Protein Hydration Shell Prevents Rapid H-D Exchange

Similar with the crystal structures of Ub (1.3 Å, PDB ID: 5DK8), R4, and R10 revealed the presence of well-defined and structured water networks throughout the protein surfaces (Figure S2), forming extensive protein-water HBs and an interconnected meshwork of structured water molecules. Previous terahertz spectroscopy revealed that the rigidity of the Ub hydration shell extending to 18 Å is sensitive to mutations, with a V26A replacement resulting in a flexible side-chain that promoted more bulk water-like dynamic hydration.^32^ The structured hydration shell surrounding R4 and R10 may enhance the protein’s resistance to thermal and chemical denaturation, acting as a protective shield. Additionally, the rotational correlation time (τ_c_) that is sensitive to molecular weight and shape, which we derived using ^15^N NMR relaxation parameters, increased from 4.15 ns for Ub to 5.2 ns for R4 and 5.5 ns for R10. Since the τ_c_ of Ub is consistent with that of a previously published report (4.1 ns),^33^ this elevated τ_c_ for the UbVs likely reflects an increased water-associated weight for R4 and R10 within the similar hydrodynamic radii (*R*_H_) derived from NMR DOSY. This apparent discrepancy can be rationalized by considering that τ_c_ is sensitive to local frictional coupling with the hydration layer,^34,35^ whereas *R*_H_ primarily reflects the overall molecular envelope. The increased τ_c_ therefore likely arises from enhanced solvent–protein interactions within a more structured hydration shell rather than a true increase in hydrodynamic size or the presence of oligomeric fractions. Accordingly, we hypothesized that R4 and R10 are encapsulated by more structured water than Ub is, resulting in a more rigid or structured hydration shell that is resistant to heat and chemical stress and engenders a slower molecular tumbling time. To validate this hypothesis, we performed NMR hydrogen–deuterium exchange (HDX) experiments over 48 h. Since the structure and HB strengths of Ub, R4, and R10 are identical (Figure 1f), we anticipated that the HDX rates at the residue level of the three proteins would be indistinguishable. Instead, the HSQC spectra of Ub, R4, and R10 (Figures 2b and S6b,c) exhibited strikingly different HDX patterns inconsistent with the HB profiles derived from their NMR Δδ_NH_/Δ*T* values. Comparison of peak intensities across the entire proteins between the 48 h D_2_O sample and the control revealed that 27 and 32 residues of R4 and R10, respectively, displayed >20% signal remaining, whereas Ub only had 12 such slowly exchanging residues (Figure 2c). Moreover, the slowly exchanging residues in Ub were primarily located in the β1, β2, and α1 regions, whereas both R4 and R10 exhibited global protection (Figure 2c). This enhanced hydrogen exchange protection coupled with the substantially increased τ_c_ values supports the existence of a structured water shell around the ProteinMPNN-designed variants, rendering them more resistant to D_2_O diffusion.

### Water Shields AI-Designed R4 from a Chemical Denaturant

Our HDX results demonstrate that a water shell surrounds the ProteinMPNN-designed variants. We hypothesized that water-crafted R4 could potentially provide exceptional protection against harsh chemical denaturation by 8 M urea. Unlike the partial or complete unfolding of Ub observed at pH 6.3 or 3.0, respectively, in the presence of 8 M urea, we detected that R4 remains extraordinarily stable in its native conformation under these denaturing conditions (Figures 3a, S3 and S4). Unexpectedly, temperature-dependent CD analysis showed that R4 maintains its characteristic secondary structure even under a combination of thermal and chemical denaturing conditions (Figure 3b). Furthermore, NMR Δδ_NH_/Δ*T* assessment of HB strengths in R4 at pH 6.3 in 8 M urea revealed a strikingly similar pattern to its native state, with only a slight reduction in strength under those denaturing conditions (Figure 3c). Impressively, even when challenged with a pH of 3.0 plus 8 M urea, the HB network in R4 remained resolutely intact, with only a 4–6 ppb/K decrease in Δδ_NH_/Δ*T*, indicative of only a slight reduction in HB stability under these extreme stress conditions (Figure 3d). Moreover, R4 remained folded in its native conformation and proved more resistant than Ub at pH 3.0 in 8 M urea. These well-evidenced outcomes highlight the astoundingly enhanced resistance of water-crafted R4 to chemical denaturation (acid and/or urea).

**Figure 3:**
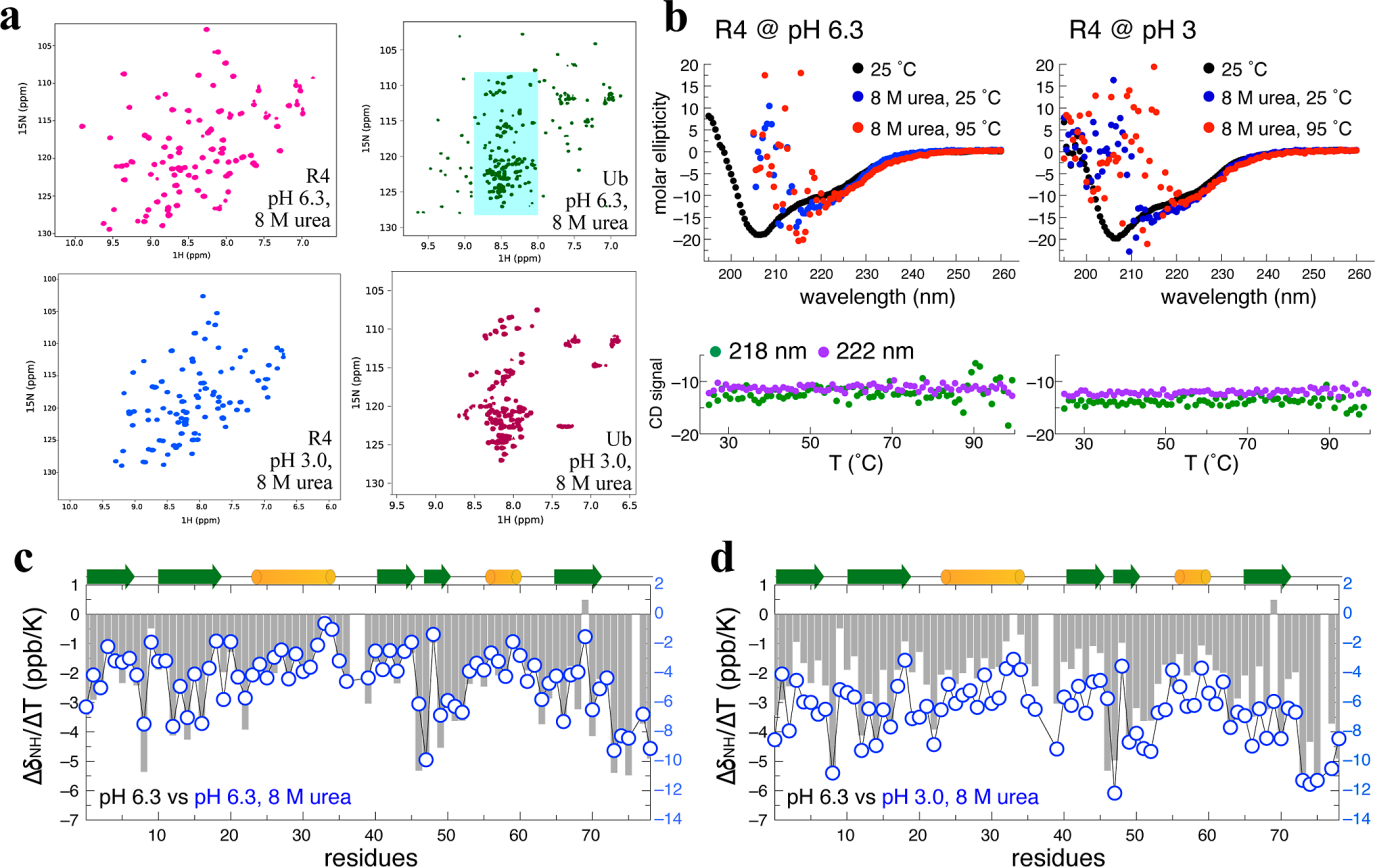
A mesostructured water shell protects R4 from urea denaturation (a). HSQC spectra of R4 in 8 M urea at two pH values (6.3 and 3.0) and at 300 K exhibit well-dispersed cross-peaks, indicating that R4 remains folded. In contrast, Ub is fully unfolded at pH 3.0 in 8 M urea and partially unfolded at pH 6.3 in 8 M urea. The cyan-shaded area highlights the unfolded cross-peaks of Ub. (b) The CD spectra of R4 in two urea-denaturing conditions at 25 °C closely resemble those recorded at the same pH without 8 M urea. When heated to 95 °C, the CD spectra remain unchanged, indicating structural integrity. Due to the strong absorbance of urea, signals at 190–215 nm are noisy. CD ellipticity at 218 and 222 nm remains constant from 25 to 95 °C under both urea-denaturing conditions. (c) NMR temperature coefficients (Δδ_NH_/Δ*T*) of R4 at pH 6.3 in the absence or presence of 8 M urea are represented by gray bars and open blue dots, respectively. The right *Y*-axis scale corresponds to the Δδ_NH_/Δ*T* values of R4 in 8 M urea at pH 6.3. (d) Similar to panel c, open blue dots represent R4 at pH 3.0 in 8 M urea, with values corresponding to the right *Y*-axis.

Remarkably, despite the harshest chemical conditions we tested, i.e., 8 M urea plus pH 3.0, the R4 variant maintained a well-behaved solution state and remained readily stable 8 months after the NMR characterization. Intrigued by this finding, we set out to conduct a thorough NMR structural analysis of R4 under these denaturing conditions. The chemical shifts of two selected leucine residues located in the β2 and α1 structural regions, L15 and L24, respectively, displayed persistent ^13^C^α^ and ^13^C^β^ chemical shifts across the pH range (6.3 to 3.0) in 8 M urea, implying unchanged structural features (Figure 4a). Secondary structures predicted by TALOS-N^36^ indicate that R4 is structurally identical under all tested conditions (Figure S4b). This unequivocal observation provides compelling evidence that the native-like structure of R4 is firmly preserved, even when challenged with severe chemical denaturation. The 3D NOESY spectra of R4 further illustrate identical long-range interactions between L67 in β5 and Y4, V5, and K6 in β1, in either native or denaturing buffer (Figure 4b), supporting native-like conformations under both conditions. In stark contrast, a previous study reported that Ub exhibited significant structural unfolding under these same challenging conditions.^37^ The NOE-restrained ensemble structures of R4 under the two denaturing conditions (8 M urea at pH 6.3 or 3.0) (Figure 4c) overlap well with the crystal structure of R4, with the backbone RMSD being within 1.1 Å. Notably, the ^15^N–R_2_ rates of R4 under these two urea-containing conditions reveal identical rigidity patterns (Figure S4a), with the ^15^N–R_2_ rate of R4 at pH 6.3 being greatly increased relative to Ub (Figure 2a). The increased ^15^N–R_2_ rate of R4 in 8 M urea solution is likely associated with increased viscosity (1.53 or 1.39 mPa·s for neutral or acidic buffer, respectively). Moreover, NMR-HDX of R4 under the two denaturing conditions also uncovered that >30% of crosspeaks were not fully exchanged from the NH to ND state 48 h after the exchange reaction (Figure 4d,e). For example, R4 at pH 6.3 plus 8 M urea presented 23 residues with >20% peak intensity remaining. These slowly exchanging residues are consistent with those in the native state (Figure 2c), indicating that exposure to 8 M urea had merely disrupted the intramolecular HBs and 3D conformation. More strikingly, the acidic denaturing condition elicited intense NH signals across the R4 sequence. More than 32 residues in R4 retained >60% NH signals 48 h after being exchanged to 100% D_2_O at pH 3.0 and 8 M urea. The resulting stronger signals could be attributable to the slower intrinsic NH-ND exchange rate at acidic pH, though the 8 M urea denaturing condition only exerted a subtle impact on the HD-exchange rate, resulting in the well-preserved NH signals of R4 at pH 3.0 in 8 M urea (Figure 4e). Together, these results are a clear indication of the robust protection provided by the crafted hydration shell surrounding the R4 structure, underscoring its extraordinary solvent resilience in contrast to native Ub (Table S2).

**Figure 4:**
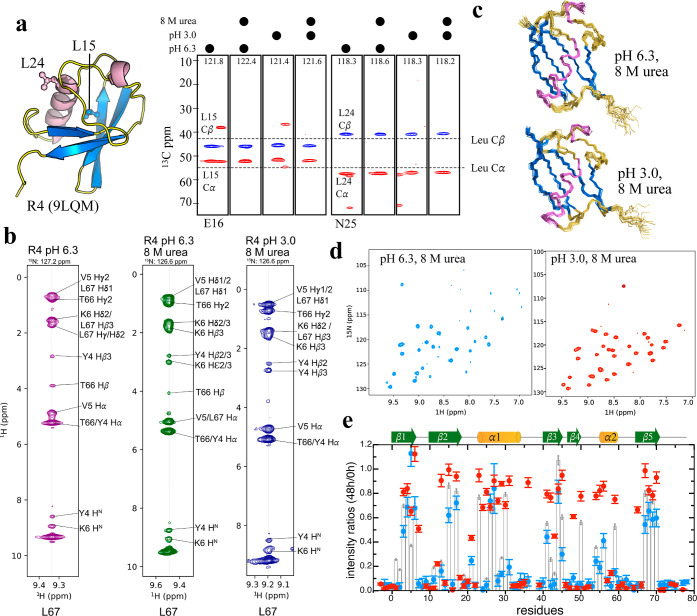
R4 remains well-structured in the urea-denaturing condition and is protected by a hydration shell (a). Hydrophobic residues L15 and L24, located in β2 and α1, were selected to illustrate ^13^Cα and ^13^Cβ chemical shifts in CBCACONH spectra (strips of E16 and N25) under four different conditions. The ^15^N plane frequencies are indicated at the top of each strip. Dashed lines represent the ^13^Cα and ^13^Cβ chemical shifts of leucine in the random-coil state. (b) Selected strips of the ^15^N-edited 3D NOESY-HSQC spectra of L67 in R4 under pH 6.3, pH 6.3 with 8 M urea, and pH 3.0 with 8 M urea, illustrating significant conservation of structural interactions from the HN of L67 to the side-chains of long-range residues, including Y4, V5, and K6. The ^15^N frequencies of L67 in the NOESY spectra are noted above each strip. (c) NOE-derived 20-mer ensemble structures of R4 at pH 6.3 with 8 M urea (top) and at pH 3.0 with 8 M urea (bottom) are shown following the same color codes presented in (a). (d) The ^15^N-HSQC spectra of R4 in 8 M urea, recorded 48 h after HDX, reveal numerous unchanged or slowly exchanged cross-peaks, indicating structural protection. (e) The intensity ratios of 48 to 0 h cross-peaks of R4 under two urea-denaturing conditions are plotted using the same color scheme as in panel (d). At pH 3.0 with 8 M urea, R4 retains >60% of signal intensity for many residues across the entire protein.

### ISG15-CTD Variants Further Demonstrate Stability Derived from Mesostructured Water

To further validate the effect of mesostructured hydration on ProteinMPNN-designed stability, we selected the C-terminal domain of ISG15 (ISG15-CTD, residues 77–157) as a second model system (Figure S7). ISG15-CTD adopts a ubiquitin-like fold (RMSD = 0.9 Å) but exhibits substantially lower thermal stability, with a *T*_m_ approximately 35 °C lower than that of Ub. Using the ISG15-CTD crystal structure (PDB ID: 6XA9, chain B) as the template, 500 ISG15-CTD variants (ICVs) were generated with ProteinMPNN. After AlphaFold structure predictions, 30 top-ranked sequences with the highest pLDDT scores were selected for protein expression and biophysical validation (Figure S7a).

All 30 ICVs exhibited >60% sequence variation relative to ISG15-CTD, and one representative, ICV-68, was confirmed to be monomeric in solution (Figures S7b,c and Table S3). Twenty ICVs with high purity were subjected to differential scanning fluorimetry (DSF), which revealed extreme thermostability in contrast to ISG15-CTD (*T*_m_ = 52.4 °C) (Figure S8). Nine ICVs were further analyzed by CD spectroscopy, all retaining well-defined secondary structures at 95 °C, whereas ISG15-CTD was fully denatured (Figure S9). DSC was then performed on five of the most stable variants (ICV-68, 84, 116, 188, and 318), revealing *T*_m_ values exceeding 120 °C for all except ICV-116, which began to unfold at 110 °C (Figure 5a). Together, these results confirm that ProteinMPNN can generate well-behaved, exceptionally stable variants from a relatively unstable template.

**Figure 5:**
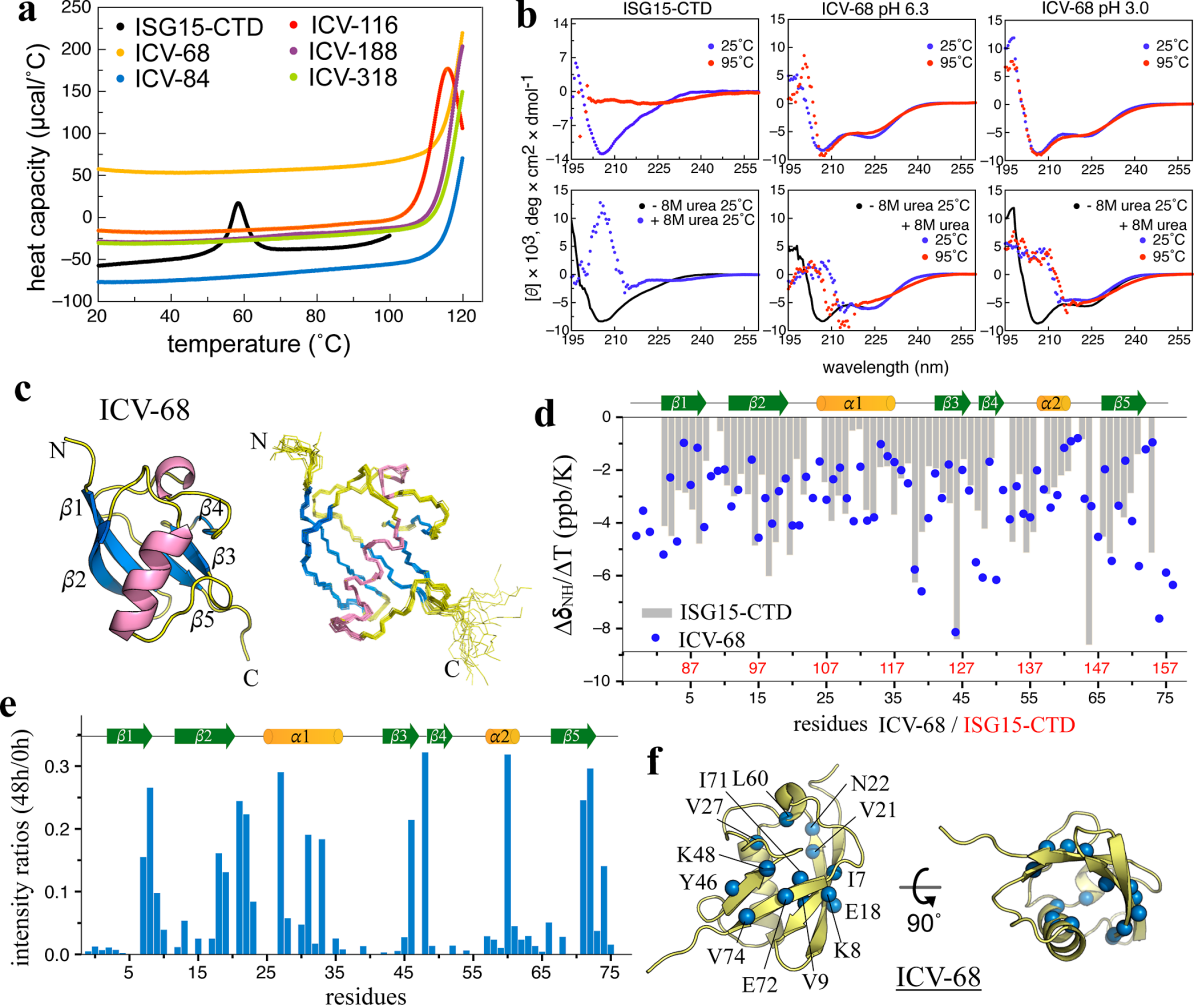
ProteinMPNN-designed ISG15-CTD variants exhibit extreme thermostability and preserved hydration-driven stability. Validation of ProteinMPNN-designed ICVs demonstrating generalized stability conferred by mesostructured hydration. (a) DSC profiles of five ICVs (68, 84, 116, 188, and 318) showing no detectable melting transitions up to 120 °C, except ICV-116 (*T*_m_ ≈ 110 °C). (b) Far-UV CD spectra of ICV-68 recorded at pH 6.3 and pH 3.0, ±8 M urea, at 25 and 95 °C, revealing complete retention of secondary structure under all conditions. (c) NMR solution structure of ICV-68 exhibiting a well-defined ubiquitin fold, with a backbone RMSD of 0.7 Å across the 20-member ensemble. (d) Temperature coefficients Δδ_NH_/Δ*T* of ICV-68 (blue dots) compared with ISG15-CTD (gray bars), indicating conserved hydrogen-bonding patterns. Residue numbers of ISG15-CTD and ICV-68 are shown on the *x*-axis in red and black, respectively. (e) HDX-NMR analysis of ICV-68 showing strong protection of backbone amides after 48 h of exchange, consistent with a rigid, solvent-ordered fold. (f) Structural representation of ICV-68 highlighting residues with >10% preserved NH signals after 48 h of HDX, illustrating localized protection mediated by mesostructured hydration. Collectively, these data demonstrate that ProteinMPNN can redesign even moderately stable templates into hyperstable variants through enhanced hydration structuring.

Given the remarkable heat, acid, and urea resistance of R4 and R10, we next examined whether the ICVs exhibited similar properties. ICV-68 remained soluble and folded under all tested conditions, including pH 6.3 and pH 3.0 with or without 8 M urea, while ISG15-CTD was fully denatured (Figure 5b). Notably, ICV-68, like R4 and R10, maintained its native conformation at 95 °C in 8 M urea at pH 3.0, demonstrating exceptional structural resilience. The NMR solution structure of ICV-68 at pH 6.3 (Figure 5c) revealed a canonical ubiquitin-like fold, with a backbone RMSD of 1.0–1.3 Å to ISG15-CTD across 20 conformers. The temperature coefficients (Δδ_NH_/Δ*T*) of ICV-68 closely match those of ISG15-CTD (Figures 5d and S10), and NMR-HDX experiments further confirmed slower amide exchange rates (Figures 5e and S11). More than 15 residues in ICV-68 retained >10% signal intensity after 48 h, whereas ISG15-CTD underwent nearly complete exchange within 4 h.

Collectively, these biophysical and NMR data demonstrate the powerful capability of ProteinMPNN to enhance protein stability through surface hydration remodeling. The designed variant ICV-68 exhibits an increase in thermal stability of over 60 °C compared to its ISG15-CTD template.

### Visualization of Protein-Water and Water–Water Interactions

Compared with Ub (11 positive/11 negative), the ProteinMPNN-designed variants contain more charged residues: R4 (15/16) and R10 (12/15) (Figure 1e). The distinct surface electrostatic potentials of the three proteins (Figure 6a) and ICVs (Figure S12), particularly for the α1 and β-strand regions, imply that the new positively and negatively charges on the surface residues of R4 and R10 may facilitate enhanced hydrogen bonding with the solvent, resulting in a well-ordered protein–solvent interaction. Consequently, R4 and R10 are less perturbed by thermal and chemical denaturation, leading to their observed enhanced protein stability.

**Figure 6:**
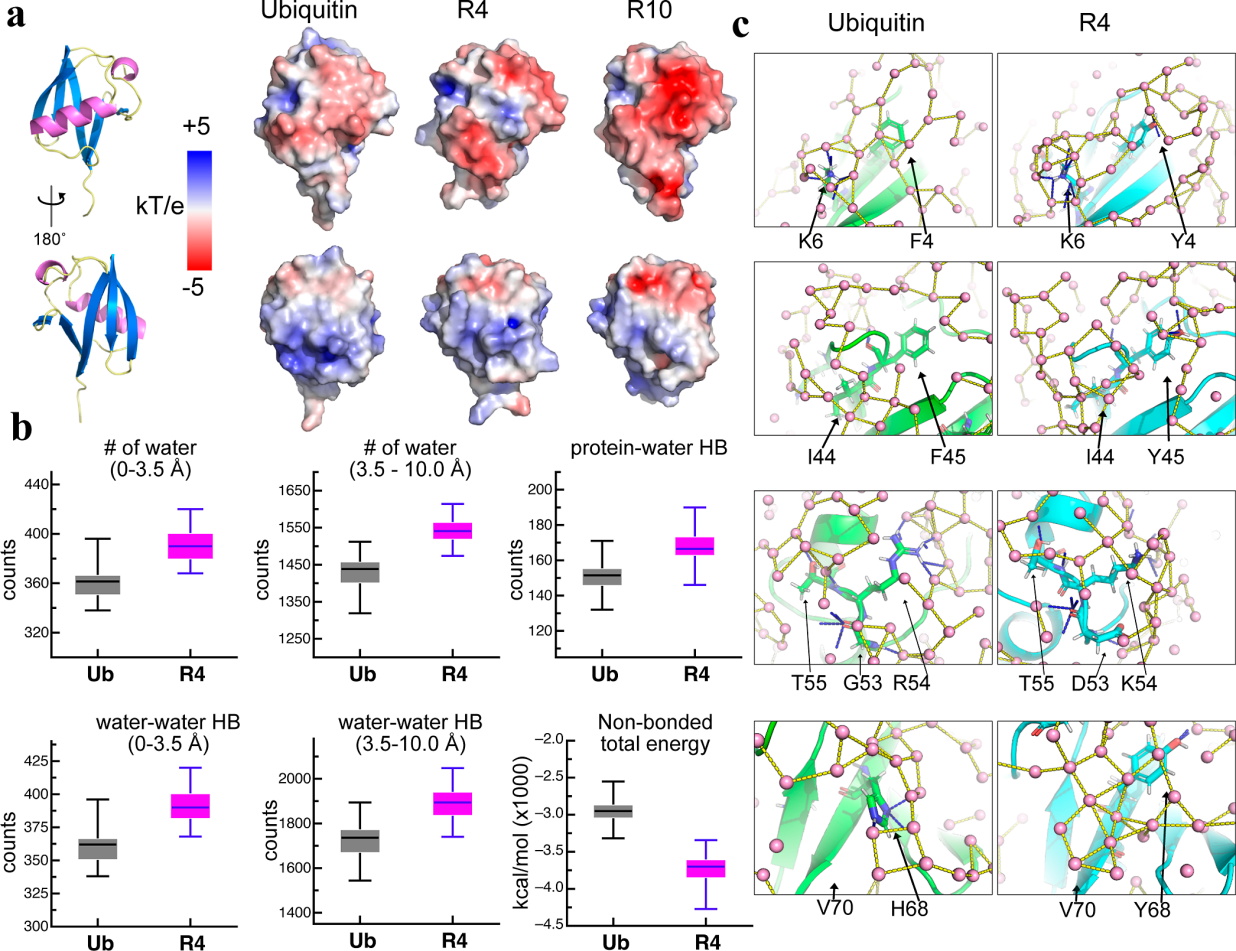
MD simulations illustrate protein-water and water–water hydrogen bonding networks (a). Surface electrostatic potentials of Ub, R4, and R10 are shown, along with their corresponding structural regions. (b). Box plots comparing hydration and interaction metrics for Ub (gray) and R4 (magenta) during MD simulations. The top row examines the hydration environment, showing the number of water molecules in the primary (0–3.5 Å) and secondary (3.5–10.0 Å) hydration shells, as well as the total number of protein–water hydrogen bonds. The bottom row focuses on water-mediated interactions, presenting the number of water–water hydrogen bonds near the protein in both hydration shells, along with the total nonbonded interaction energy between the protein and water. (c) Protein–solvent and water–water HBs in the primary hydration shell are colored in blue and yellow dashed lines, respectively. Water molecules are shown as pink spheres. Residues in Ub (left) and R4 (right) were selected to present the HB networks corresponding to the NMR HDX data shown in Figure 2.

We conducted molecular dynamics (MD) simulations to directly visualize the profound impact of water on the stability of the ProteinMPNN-designed variants. These simulations revealed that the water molecules within 3.5 Å (named the primary hydration shell)^38^ of R4 and R10 exhibited substantially increased residence times compared to their counterparts in Ub (Figures 6b and S13). The MD simulations confirmed that the slowly NH-ND exchanging residues (see Figure 2c) are networked by increasing the protein–solvent and water–water HBs. Notably, the F4Y, F45Y, H68Y, G53D, and R54K residues altered from Ub to R4 are consistently hydrogen bonded to water through side-chains, as well as each residue having more water–water HBs surrounding them (Figure 6c). Furthermore, the secondary hydration shell (i.e., between 3.5 and 10 Å of the protein surface)^39^ was also informative, as both R4 and R10 retained a greater number of water molecules across the residence times than the respective environment of Ub. For instance, R4 interacts with water molecules on average 40 and 100 times more in the primary shell and secondary shell, respectively, than Ub. A detailed characterization uncovered that the water retained in the primary hydration shell of R4 and R10 enabled more extensive (∼5–10%) protein–solvent HBs than possible for Ub. Consequently, the ProteinMPNN-designed variants exhibit lower nonbonded interaction energy between the protein and solvent, which implies better aqueous solubility and stability than native protein. Additionally, the water molecules within the hydration shells of the ProteinMPNN-designed proteins displayed stronger orientational order through increased water–water HBs, resulting in reduced mobility compared to bulk water. Together, these scenarios strongly support that these ProteinMPNN-designed proteins are crafted by mesostructured water molecules, so that R4 displays a slower molecular tumbling time than Ub.

The combined experimental and computational results demonstrate that mesostructured water molecules protect the ProteinMPNN-designed variants from denaturation induced by heat, acid, or chemical denaturants. Because R4 and R10 exhibit extreme thermal resistance with melting temperatures exceeding 120 °C, molecular dynamics simulations at elevated temperature (500 K) were performed to further assess their stability relative to wild-type ubiquitin. As shown in Figure 7a, the radius of gyration (*R*_g_) of R4 and R10 remain nearly constant (∼12 Å) throughout the 500 ns simulations, whereas the *R*_g_ of Ub begins to increase after ∼100 ns, reaching >25 Å between 200 and 300 ns before partially collapsing to ∼15 Å. This pronounced fluctuation in *R*_g_ suggests extensive structural instability and reorganization of Ub at high temperature. Representative snapshots of the trajectories illustrate that Ub undergoes unfolding and partial collapse (Figure 7b). In contrast, both R4 and R10 maintain their compact native conformations throughout the 500 ns simulations, with negligible deviation between the initial and final structures (Figure 7c), consistent with their exceptional thermal stability.

**Figure 7:**
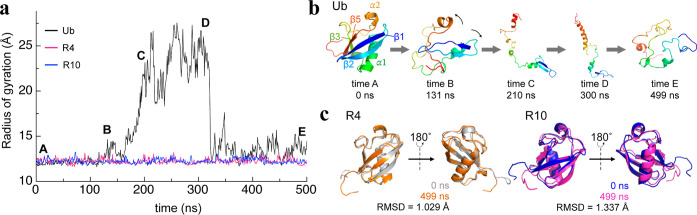
High-temperature MD simulations demonstrate the structural persistence of ProteinMPNN-designed variants. High-temperature MD simulations reveal exceptional thermal stability of ProteinMPNN-designed variants. (a) Time evolution of the *R*_g_ for ubiquitin, R4, and R10 during 500 ns simulations at 500 K. Ubiquitin shows progressive expansion and collapse after 100 ns, consistent with unfolding and misfolding events, whereas R4 and R10 maintain nearly constant *R*_g_ values (∼12 Å) throughout the simulation. (b) Representative structural snapshots of ubiquitin at selected time points illustrating gradual unfolding and loss of tertiary packing. (c) Superimposed structures of R4 and R10 at 0 and 499 ns demonstrating minimal deviation from the initial conformations. The results indicate that reinforced mesostructured hydration effectively preserves the native fold of the ProteinMPNN-designed variants under extreme thermal stress.

## Discussions

The ProteinMPNN-designed Ub variants R4 and R10 demonstrate extraordinary resilience against thermal and chemical denaturation, surpassing the stability of Ub. This enhanced robustness is directly linked to the formation of a mesostructured water shell, which acts as a protective barrier against harsh environmental conditions. Our MD simulations reveal that these structured water molecules are tightly bound and less accessible to solvents, contributing significantly to the increased stability observed in our hydrogen–deuterium exchange experiments (Figure 8a).

**Figure 8:**
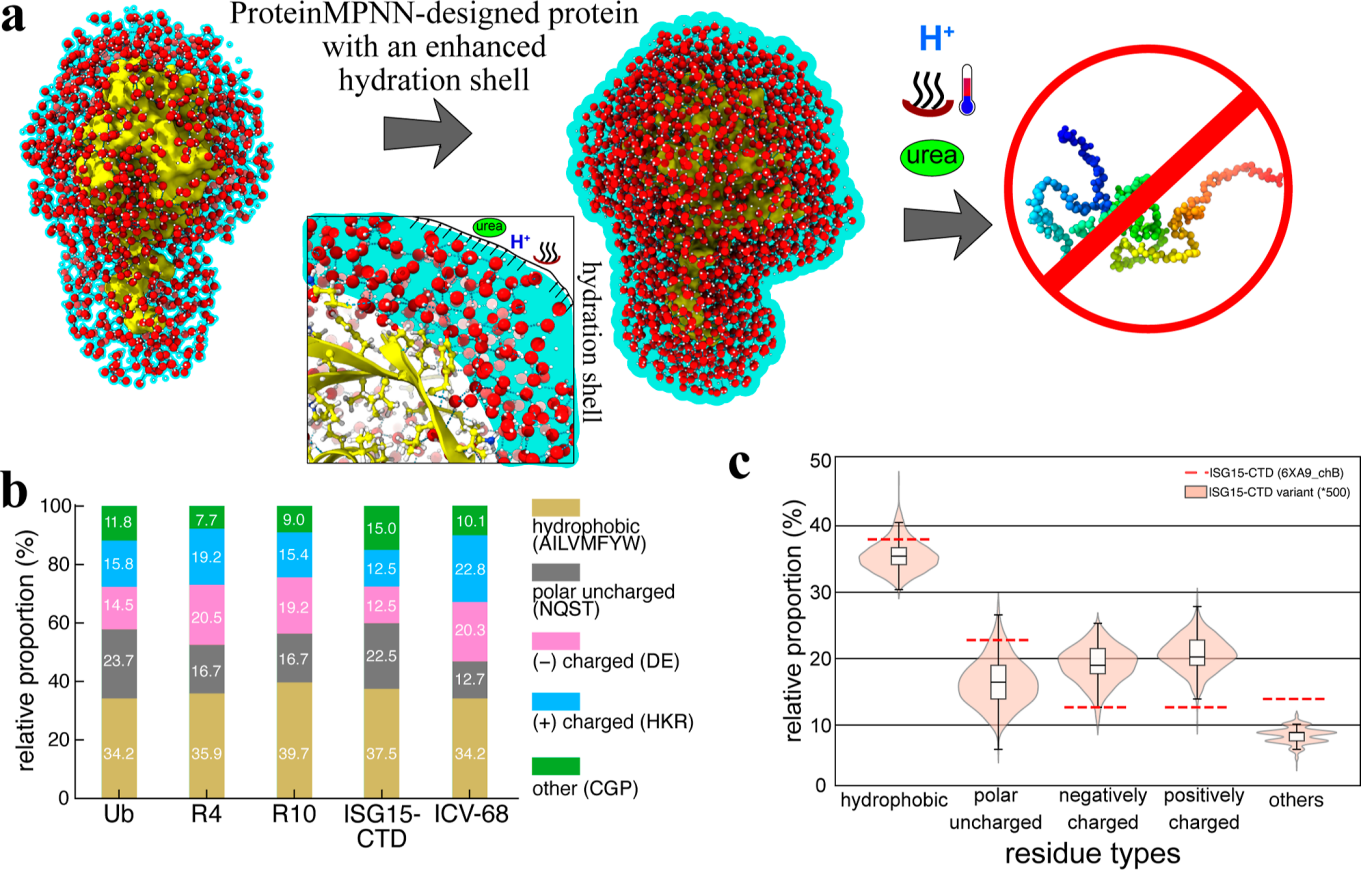
ProteinMPNN enhances stability by optimizing hydration water networks (a). Ub exhibits high thermal stability due to an extensive hydration shell extending up to 18 Å, conferring significant heat resistance. However, exposure to a combined acidic solvent and urea environment leads to complete unfolding. In contrast, the AI-designed UbV mimics Ub in structure, but features a more ordered mesostructured hydration shell. This enhanced hydration network provides superior insulation, rendering the UbV highly resistant to heat, urea, acidic conditions, and even combined stress environments. As a result, the UbV demonstrates exceptional stability under extreme conditions. (b), Amino acid composition of ubiquitin, R4, R10, ISG15-CTD, and the ProteinMPNN-designed variant ICV-68. Both positively and negatively charged residues are increased in the designed variants compared with their respective templates. (c) Statistical analysis of 500 ProteinMPNN-designed ISG15-CTD variants (ICVs) showing consistent enrichment of charged residues, indicating that ProteinMPNN preferentially introduces surface charge across designs. Comparative electrostatic surface maps of nine representative ICVs and ISG15-CTD are shown in Figure S12.

Beyond R4 and R10, six additional ProteinMPNN-designed Ub variants and 30 ISG15 CTD variants (ICVs), each with over 30 sequence alterations, have also been confirmed as exhibiting great thermal stability (Figures S1b,c and S10). Consequently, ProteinMPNN can readily generate highly stable proteins even with extensive residue replacements.^20–22^ Since ProteinMPNN did not alter the key residues forming the hydrophobic core (Figure 1e), most substitutions occur at surface-exposed positions. We found that these surface residues are frequently replaced by charged amino acids. For example, R4 contains approximately 6% more negatively charged and 3.5% more positively charged residues than wild-type Ub, while ICV-68 exhibits 7.8% and 10.3% increases, respectively, relative to ISG15-CTD (Figure 8b). A broader statistical analysis of 500 designed ISG15-CTD variants (ICVs) further revealed that both positive and negative charges are enriched on average by 8–10% (Figure 8c).

The comparative sequence analysis of Ub, R4, R10, ISG15-CTD, and ICV-68 indicate that ProteinMPNN consistently introduces additional charged residues, thereby increasing the overall surface polarity of the variants. Electrostatic potential maps further show that these new charges form broader, more contiguous regions on the protein surface, favoring retention and ordering of interfacial water molecules. Together, these data suggest that the reinforced hydration shell in the designed variants arises primarily from an enriched distribution and clustering of surface charges that promote stronger protein–water hydrogen bonding and long-range water–water connectivity. The resulting charged side-chains could attract more water molecules and potentially enhance hydration. In addition to increasing the number of surface-exposed charged residues, ProteinMPNN appears to reorganize surface electrostatics in a way that favors the formation of structured hydration shells. The resulting network of solvent-coupled hydrogen bonds likely underlies the exceptional resistance of the designed variants to heat, acid, and chemical denaturation. This optimal charge distribution likely produces a synergistic effect, leading to a measurable 10% increase in water density and enhanced solvent-associated HB networks around the protein. Although dissecting the precise contribution of individual mutations remains challenging, the collective sequence and electrostatic features provide a mechanistic explanation for the formation of mesostructured hydration shells and the extraordinary stability of the ProteinMPNN-designed proteins.

While hydration dynamics have long been recognized as central to protein stability, the specific sequence patterns that promote persistent or structured hydration remain incompletely defined. Our analyses reveal that ProteinMPNN tends to enrich charged and polar residues in clustered surface regions, producing continuous electrostatic landscapes that favor cooperative solvent ordering. Such charge patterning likely enables denser and longer-lived water networks, bridging neighboring residues through solvent-mediated hydrogen bonds. This finding highlights a potential design principle for physics-guided and hybrid AI–physics protein engineering: strategically distributed surface charges and polar patches can tune hydration structuring to achieve exceptional resilience without altering the hydrophobic core.

Our findings establish that the redesign of Ub by the ProteinMPNN algorithm enhances protein stability through an unappreciated mechanism, i.e., harnessing structured hydration, rather than merely by optimizing internal hydrophobic interactions. This study underscores the power of computational protein design to generate highly resilient biomolecules with engineered water-crafted properties. These insights pave the way for the rational design of proteins with enhanced stability, unlocking a mechanism by which stable and extremely durable proteins can be created for biotechnological and therapeutic applications.

## Materials and Methods

### Protein Preparation and Purification

Ubiquitin, ISG15 CTD (77–157), and its variants designed by ProteinMPNN^20^ were prepared as described previously^21^ or followed the same procedure. Thirty ICVs were selected for experimental validations as the evidence of the second example. In summary, the hexahistidine-tagged proteins were eluted from a nickel affinity column, followed by TEV protease digestion to remove the tag. The tag-free proteins were further purified using size exclusion chromatography on a Superdex 75 increase 10/300 GL column with an ÄKTA FPLC system. The pure fractions were then concentrated to 5–25 mg/mL, aliquoted, snap-frozen in liquid nitrogen, and stored at −80 °C. For NMR analysis, the proteins were expressed in M9 medium supplemented with ^15^N ammonium chloride and/or ^13^C glucose as the sole nitrogen and carbon sources, respectively. The purification and storage procedures for the isotope-labeled proteins were identical to those used for the unlabeled samples.

### Crystallization and Structure Determination

Sitting drop vapor diffusion was used to crystallize R10 at 22 °C by mixing equal volumes of R10 solution (10, 15, or 20 mg/mL) and screening solution. The initial crystal was obtained in a condition containing 0.2 M MgSO_4_ and 10% PEG4000, which was further optimized to 0.2 M MgSO_4_ and 8% PEG4000 for data collection. We added 11% glycerol to the crystallization solution as a cryoprotectant. X-ray diffraction data for the R10 crystal were collected at the Taiwan Photon Source 07A beamline at the National Synchrotron Radiation Research Center (NSRRC TPS-07A). Eleven data sets were collected at resolutions of between 1.4 and 1.8 Å, resulting in two different space groups, i.e., *I*222 and *P*2_1_2_1_2. A total of 300–900 diffraction images were acquired with an oscillation frame rate of 0.2–0.3°. The diffraction data were processed using HKL2000.^40^ The phase was determined by molecular replacement using the R10 structure predicted by AlphaFold2, and the structure of a 1.55 Å data set was iteratively refined and visualized through Phenix^41^ and Coot,^42^ respectively.

The R4 crystal was prepared using methods similar to those applied in previous studies,^21^ with modifications such as a lower R4 concentration, varied pH buffer, and an increased drop size from 2 to 4 μL in the sitting drop vapor diffusion setup. The R4 crystals were grown at 22 °C under conditions containing 2.4 M (NH_4_)_2_SO_4_, 0.1 M sodium citrate at pH 3.7, and 2% MPD, and were then harvested in the same buffer with 4.6% MPD. X-ray diffraction data for the R4 crystal were collected at the NSRRC TPS-07A beamline, employing strategies analogous to those used for the R10 crystals. Eight data sets were collected at resolutions ranging from 1.3 to 1.6 Å and processed using HKL2000 software. A 1.39 Å diffraction set was indexed and scaled in the *P*3_2_21 space group, with the phase determined by the previously published 3.0 Å structure (PDB 8J0A) and used for the final structure determination.

Crystallographic statistics are provided in Table S1 and the high-resolution structures of R4 and R10 are publicly available from the Protein Data Bank, with accession codes 9LQM and 9LQK, respectively.

### NMR Spectroscopy, Data Analysis, and Structure Determination

The NMR experiments for Ub, R4, R10, ISG15-CTD, and ICV68 were conducted on Bruker NEO spectrometers equipped with TCI or TXO cryogenic probes operating at 850 or 600 MHz, respectively, housed in the high-field NMR center at Academia Sinica. Backbone and side-chain assignments were obtained using standard 3D triple-resonance experiments at 300 K with 1.0–1.4 mM uniformly ^15^N- and ^13^C-labeled proteins. To reduce data collection time, nonuniform sampling and reconstruction methods were employed. Specifically, a Poisson-Gap sample schedule with 10% or 20–30% data points was applied for 3D resonance or 3D NOESY experiments, respectively, and the data were processed and reconstructed using the hmsIST algorithm.^43^ NMR samples were prepared at pH 6.3, pH 3.0, or in 8 M urea at the same pH and NaCl concentration, with buffer exchange applied to R4 for the acidic or urea conditions. ^15^N-HSQC spectra were recorded at various temperatures, pH values, and time points using 0.1 mM ^15^N-labeled proteins. Data processing and analysis were performed using NMRPipe^44^ and Poky.^45^

Spectral assignments were performed using online tools such as ARTINA in NMRtist^46^ and I-PINE.^47^ The sequential assignment findings were further validated by inspecting the ^15^N-based connectivities in the 3D HNcaNNH spectrum. Automated procedures achieved an assignment accuracy of 96–98% across the eight NMR conditions, with 1–2 residues requiring manual corrections. The ^15^N-edited and ^13^C-edited 3D NOESY experiments, with mixing times of 120 ms, were assigned using CYANA^48^ and PONDEROSA,^49^ followed by manual verification.

NMR relaxation experiments were performed at 850 MHz using the Bruker pulse library. The ^15^N longitudinal (*R*_1_) and transverse (*R*_2_) relaxation rates, as well as the steady-state heteronuclear ^15^N–^1^H NOE data, were collected at 300 K. The experiments and analyses were carried out as described.^50^ Specifically, twelve relaxation time points were used for the ^15^N–R_1_ and ^15^N–R_2_ measurements, with a 3 s relaxation delay. A 5 s ^1^H saturation period was applied for heteronuclear NOE acquisition. The R_1_ and R_2_ relaxation rates were calculated by two-parameter curve fitting using Poky. The NOE values were determined from the ratio of cross-peak heights in the saturation and reference spectra. The rotational correlation time τ_c_ was calculated using tensor2.^51^

Temperature dependence were examined at the temperature range of 281–320 K in 5° increments and collecting ^1^H–^15^N HSQC spectra at each point. Chemical shift calibrations at each temperature point were performed using deuterated 3-(trimethylsilyl)-2,2,3,3-tetradeuteropropionic acid (TSP-*d*_4_) as an internal chemical shift reference at 0.0 ppm.

Hydrogen–deuterium exchange experiments were performed by dissolving lyophilized ^15^N-labeled proteins in either 100% H_2_O or 100% D_2_O resulting in a protein concentration of 100 μM, with the former serving as the control condition for the exchange experiments. ^1^H–^15^N HSQC spectra were acquired over time, with the initial spectrum collected within 5 min of dissolution in D_2_O. The 250–390 HSQC spectra (5.5 min each) of R4, R10, and Ub were acquired within 48 h of dissolution in D_2_O. HDX spectra for the urea solution were prepared by mixing lyophilized R4 powder (exchanged to H_2_O) and urea-containing buffer. The urea buffer for 100% D_2_O was prepared by dissolving lyophilized 1000 μL buffer powder in 1000 μL 100% D_2_O and this step was repeated twice to ensure the amide proton in urea had been substituted by deuterium prior to dissolving the R4 powder. The 115–130 HSQC spectra of R4 dissolved in the two 8 M urea buffers (pH 6.3 and 3.0) were then collected (23 min each). The peak intensities of amide protons were quantified and normalized against the control spectrum using NMRPipe and in-house scripts.

The NMR structures were calculated using Xplor-NIH 3.9,^52^ employing both NOE and dihedral angle restraints. The NOE-derived distance restraints were defined using CYANA, NMRtist, or PONDEROSA, followed by manual inspection. TALOS-N^36^ was utilized to determine the angular restraints. A simulated annealing protocol (fold.py) from Xplor-NIH was performed, starting with 500 initial structures. The lowest energy structure was selected for refinement, resulting in 150 structures. From this set, the 20 lowest energy refined structures were selected and aligned based on the structural region spanning residues 1–72, generating an ensemble. The quality of the structures was assessed using Molprobity,^53^ PSVS2,^54^ and the PDB validation server. The NMR structures are summarized in Table S2.

Hydrostatic pressure NMR experiments were conducted using a specialized high-pressure cell within a Bruker AVANCE III 950 MHz spectrometer at the Institute for Protein Research, Osaka University. ^15^N-HSQC spectra were acquired at 300 K under a range of pressures from 1 to 2500 bar, with data points collected at specific pressure levels (1, 30, 500, 1000, 1500, 2000, and 2500 bar). The peak intensities from these HSQC spectra were analyzed to assess any chemical shift changes and signal intensity variations across the entire 1 to 2500 bar pressure range.

DOSY experiments were performed at 300 K using a Bruker AVANCE III spectrometer equipped with a BBFO probe. The experimental parameters included a gradient length of 2 ms, a delay time of 1000 ms between the two gradient pulses, and a gradient strength that was varied linearly in 32 steps between 2% and 95% of the maximum gradient coil power. The signals from methyl groups resonating between 0.2 and 1.0 ppm were integrated to determine the translational diffusion coefficient, *D*_trans_, using Bruker Topspin 3.6.0. These DOSY experiments were performed to measure the translational diffusion of the protein samples, providing insights into their hydrodynamic size and oligomeric state in solution.

### Circular Dichroism Spectroscopy

Circular dichroism (CD) measurements were conducted using a Jasco J-815 spectrometer according to a protocol described previously.^21^ All samples, prepared at a concentration of 10 μM, were diluted in buffer and analyzed in a 1 mm quartz cuvette. Full CD spectra were recorded from 260 to 195 nm at both 25 and 95 °C under various experimental conditions. Additionally, thermal denaturation experiments were carried out, monitoring the CD signal at 218 and 222 nm over a temperature range of 25 to 95 °C with 1 °C increments. The presented CD data represent the average of triplicate measurements, acquired at a scanning speed of 50 nm/min and a digital integration time of 1 s.

### Differential Scanning Calorimetry

The protein samples were prepared by dialysis into NMR buffer using an Amicon concentrator, then diluted to 1 mg/mL and loaded onto a Malvern PEAQ-DSC system at the Biophysics Core Facility, Academia Sinica, for the stability experiments. The samples were scanned at a rate of 200 °C/h from 30 to 100 or 120 °C. All data were analyzed using MicroCal PEAQ-DSC software v1.63 to subtract the buffer scans before the individual protein runs and then spline interpolation of the baseline was applied under the thermal transition. The model-free integrated calorimetric enthalpy of the transition and the melting temperature (*T*_m_) are reported where available.

### All-Atom Molecular Dynamics Simulations

The initial structures of the AI-designed Ub variants R4 and R10 were obtained from the crystal structures, PDB IDs: 9LQM and 9LQK. Since these PDB entries are dimeric forms, the monomeric unit was extracted and used as initial structures for the MD setup. Crystal water molecules were retained during system preparation. The MD trajectory of Ub, with a two-residues extension from the C-terminus, was obtained from a previous study.^5^ To ensure consistency, both R4 and R10 were extended by two residues at the C-terminus, and extra residues from the N-terminus were removed. As a result, all tested UbVs consisted of 78 residues.

All MD simulations were performed using the AMBER20 package with a FF14SB force field.^55,56^ Missing side-chain atoms were built using *tleap* from the AMBER 20 package.^56^ Hydrogen atoms, amino acid side-chains, and the entire protein system were minimized for 500, 1000, and 5000 steps, respectively, in a generalized Born implicit solvent to avoid unrealistic side-chain interaction. The resulting minimized structures were subsequently solvated in a TIP3P^57^ water box extending 12 Å from the edge of the protein. To ensure charge neutrality, sodium counterions (Na^+^) were introduced into the simulation system. Specifically, three Na^+^ ions were added for R10, and none were required for either R4 or Ub. The solvated system contained approximately 30,000 atoms. Then, the water molecules were minimized for 1000 steps while keeping the protein restrained. This step was followed by 2000 steps of unrestrained minimization of the entire system.

Equilibration was performed under constant pressure and temperature (*NPT* ensemble) by gradually increasing the temperature from 50 to 275 K in 25 K increments, with each temperature maintained for 100 ps, before final equilibration at 298 K for 500 ps. Production simulations were carried out at 298 K using a Langevin thermostat^58^ with a 2 fs time step for 500 ns employing a 12 Å cutoff for short-range nonbonded interactions and handling long-range electrostatics via the Particle Mesh Ewald (PME) method.^59^ Raw MD trajectories were saved every 1 ps for detailed analysis. CPPTRAJ and visual molecular dynamics (VMD) were used for data analysis.^60,61^ To ensure the systems were fully equilibrated, the first 40 ns of the MD simulation were discarded, leaving 460 ns for analysis. MD trajectories were resaved every 100 ps, yielding a total of 4600 frames for further analysis.

To evaluate thermal stability, high-temperature MD simulations were performed for Ub, R4, and R10. Restart files from the equilibrated 298 K trajectories were used to continue the equilibration until 500 K. The temperature was raised in 25 K increments from 298 to 500 K, with each step maintained for 100 ps, followed by a 500 ps equilibration at 500 K. Production simulations were then carried out for 500 ns at 500 K using the same simulation protocol as the 298 K runs. Trajectories were saved every 1 ps.

### Mesostructured Water Analysis

To understand how surrounding water molecules stabilized the protein, we first quantified the number of water molecules within two defined regions, i.e., the primary hydration shell (within 3.5 Å of protein-heavy atoms)^38^ and the secondary hydration shell (between 3.5 and 10 Å).^39^ To further understand how the interaction between protein-water and water–water affects the overall stability of the protein, we quantified the number of hydrogen bond contributions in the protein-water hydrogen network and water–water hydrogen bond network using a distance cutoff of 3.5 Å and an angle criterion of 30°. Similarly, hydrogen bonds among water molecules were counted for both the primary and secondary hydration shells.

Moreover, we performed interaction energy calculations for each resaved frame to evaluate the intermolecular interactions between a protein and the surrounding water. The nonbonded interaction energies, encompassing both van der Waals and electrostatic components, were calculated using the NAMD energy calculations,^62^ with a 12 Å cutoff. To mitigate short-term fluctuations and emphasize long-term trends, cumulative averages were performed on all analyses over the 460 ns analysis window.

## Supplementary Material



## Data Availability

The crystal structures of R4 and R10 have been deposited to PDB (https://www.rcsb.org) with accession codes 9LQM and 9LQK, respectively. The NMR assigned chemical shifts of R4, R10, and R4 at pH 3.0, as well as for R4 dissolved in 8 M urea, have been deposited to BMRB (https://bmrb.io) with the accession codes 36736, 36737, 36738, and 36739. The PDB codes are 9M9G, 9M9H, 9M8X, and 9M8W. Details are summarized in Tables S1 and S2.

## Notes

Methods of SEC-MALS and DSF experiments and additional experimental data and tables (PDF)

## Abbreviations

NMR: nuclear magnetic resonance
AI: artificial intelligence
CD: circular dichroism
HB: hydrogen bond
HDX: hydrogen–deuterium exchange.
