# Supplementary material for: Mesostructured Water Enhances Stability of ProteinMPNN-Designed Ubiquitin-Fold Proteins
Source: J Am Chem Soc. 2026 Feb 5;148(7):7363–77. doi: 10.1021/jacs.5c19875 (PMC12951454; doi:10.1021/jacs.5c19875)
Supplement: Supplementary file 1 [file ja5c19875_si_001.pdf]

Supporting Information

Mesostructured Water Enhances Stability of ProteinMPNN-designed Ubiquitin-fold Proteins

Lu-Yi Chen [a,b], Wei-Lin Lu [a], Tanvi Pathania [c], I-Hsuan Chu [a], Meng-Ru Ho [a], Wei-Chen Chuang [a], Yuan-Chao Lou [d], Ta I Hung [c], Yohei Miyanoiri [e], Chia-en A Chang [c], and Kuen-Phon Wu[a,b, *]

a. Institute of Biological Chemistry, Academia Sinica, Taipei, 115, Taiwan

b. Institute of Biochemical Sciences, College of Life Science, National Taiwan University, Taipei, 115, Taiwan

c. Department of Chemistry, University of California Riverside, Riverside, CA, US

d. Biomedical Translation Research Center, Academia Sinica, Taipei, 115, Taiwan

d. Research Center for Next-Generation Protein Sciences, Institute for Protein Research, Osaka University, 3-2 Yamadaoka, Suita, Osaka, 565-0871, Japan

* E-Mail: kpwu@as.edu.tw

Table of contents

Experimental methods

Supplementary Figures S1-S13

Table S1 Data collection and structural quality of crystal structures R4 and R10

Table S2 NMR assignment and structure determination of R4, R10, and Ub

Table S3. Sequences of ISG15-CTD and the top 30 ranked variants

Supporting experimental methods

Differential Scanning Fluorimetry (DSF)

Purified protein samples were first diluted in assay buffer containing 20 mM Tris-HCl (pH 8.0) and 150 mM NaCl to reach a final concentration of 0.2 mg/mL. To prepare each reaction, 15 µL of the diluted protein solution was mixed thoroughly with 1 µL of 100× SYPRO Orange dye (Sigma-Aldrich). The mixtures were gently pipetted into a LightCycler 480 Multiwell Plate 96 (Roche) to ensure uniform distribution and minimize air bubbles, which can interfere with fluorescence readings.

Thermal shift assays were carried out using a LightCycler 480 II real-time PCR system (Roche). Excitation and emission wavelengths were set to 465 nm and 580 nm, respectively, corresponding to the optimal excitation/emission properties of SYPRO Orange. The fluorescence intensity was continuously recorded while the temperature was gradually increased from 20 °C to 95 °C at a controlled heating rate of 0.01 °C/s.

The resulting fluorescence profiles, which reflect the exposure of hydrophobic regions upon protein unfolding, were exported as melting curves. The melting temperature (T_m) for each protein was determined as the inflection point of the fluorescence transition curve, corresponding to the midpoint of thermal unfolding. All measurements were performed in triplicate to ensure reproducibility, and the data provided quantitative comparisons of thermal stability among the ISG15-CTD and ICV variants.

Size exclusion chromatography combined with multi-angle light scattering (SEC-MALS)

SEC-MALS analysis was carried out on an Agilent 1260 Infinity HPLC system connected to a Wyatt miniDAWN TREOS light scattering detector and an Optilab T-rEX refractive index detector (Wyatt Technology). The system was calibrated using bovine serum albumin (A1900, Sigma) as a molecular weight standard. Chromatographic separation was performed on an Agilent Bio SEC-3 column (300 Å, 4.6 × 300 mm) pre-equilibrated with a buffer containing 25 mM Tris-HCl (pH 7.6), 200 mM NaCl, and 0.02% NaN_3. A 100 µg sample of purified ICV-68 was loaded onto the column and eluted at a constant flow rate of 0.35 mL/min.

The elution profile was monitored simultaneously by UV absorption, light scattering, and refractive index detection. Molecular mass determination was performed using ASTRA 6 software (Wyatt Technology), applying a refractive index increment (dn/dc) of 0.185 mL/g. The theoretical molecular weight of ICV-68 with extra GGS at the N-terminus is 9.2 kDa. The analysis revealing 9.25 kDa confirmed the monomeric state and solution homogeneity of ICV-68 under the tested conditions.

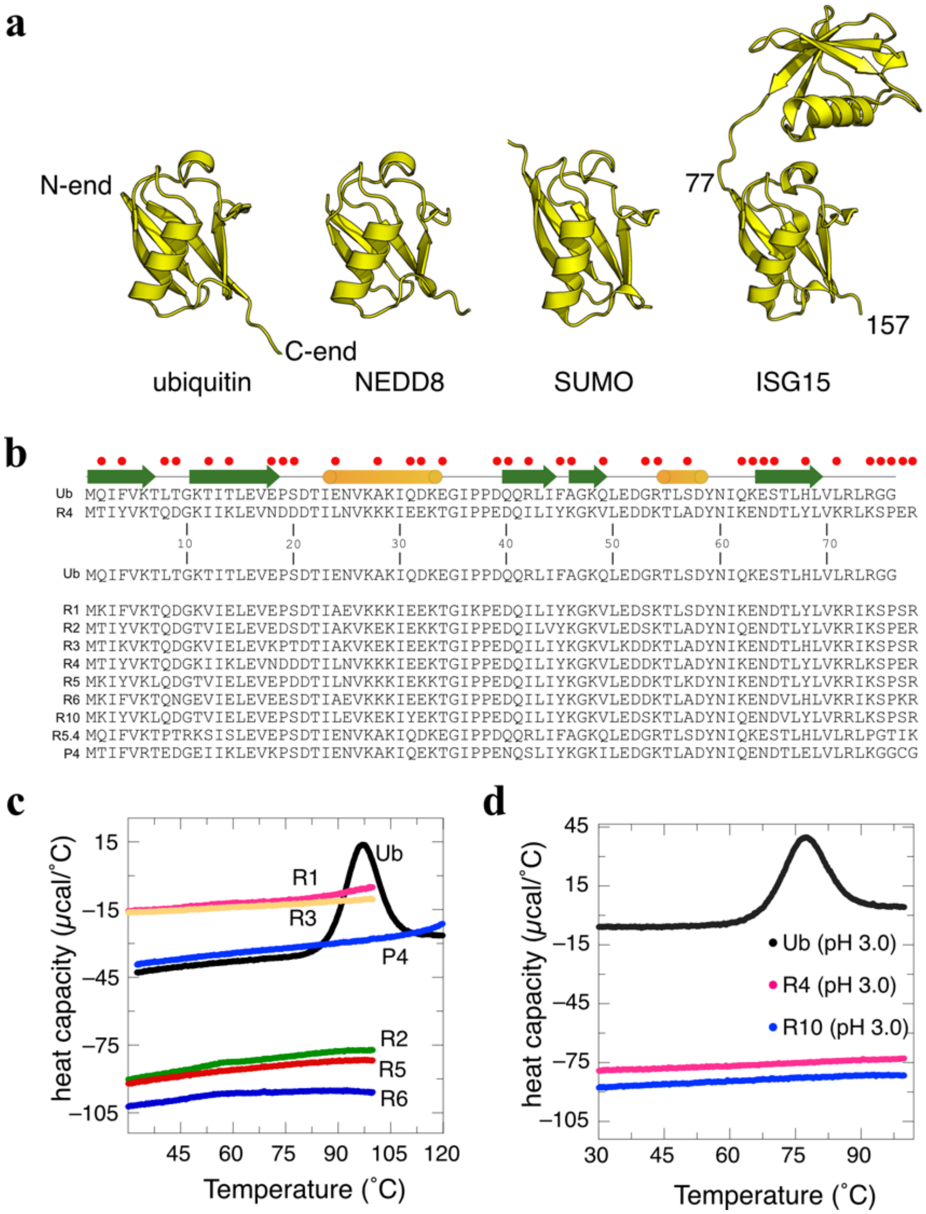

Figure S1. Stability of ubiquitin-like proteins and their variants
(a). Biological ubiquitin-like proteins, including ubiquitin, NEDD8, SUMO, and ISG15, are aligned and presented, showcasing their consistent conformation and structural regions. ISG15 is composed of two ubiquitin-fold domains, with the C-terminal domain residues 77-157 being aligned with those of the other proteins. (**b**)**.** Sequence alignment of ubiquitin and ubiquitin variants, with secondary structural regions indicated. The red dots denote the mutations in R4 compared to Ub. **(c).** Differential scanning calorimetry (DSC) heat capacity profiles of Ub and six other ProteinMPNN-designed ubiquitin variants, exhibiting minimal changes compared to the transition peak of Ub. **(d).** Unlike for Figure **1b**, the DSC experiment was performed at pH 3.0 for Ub, R4, and R10, with R4 and R10 remaining well-folded upon heating to 100 °C, supporting a stable conformation at pH 3.0 and high temperatures.

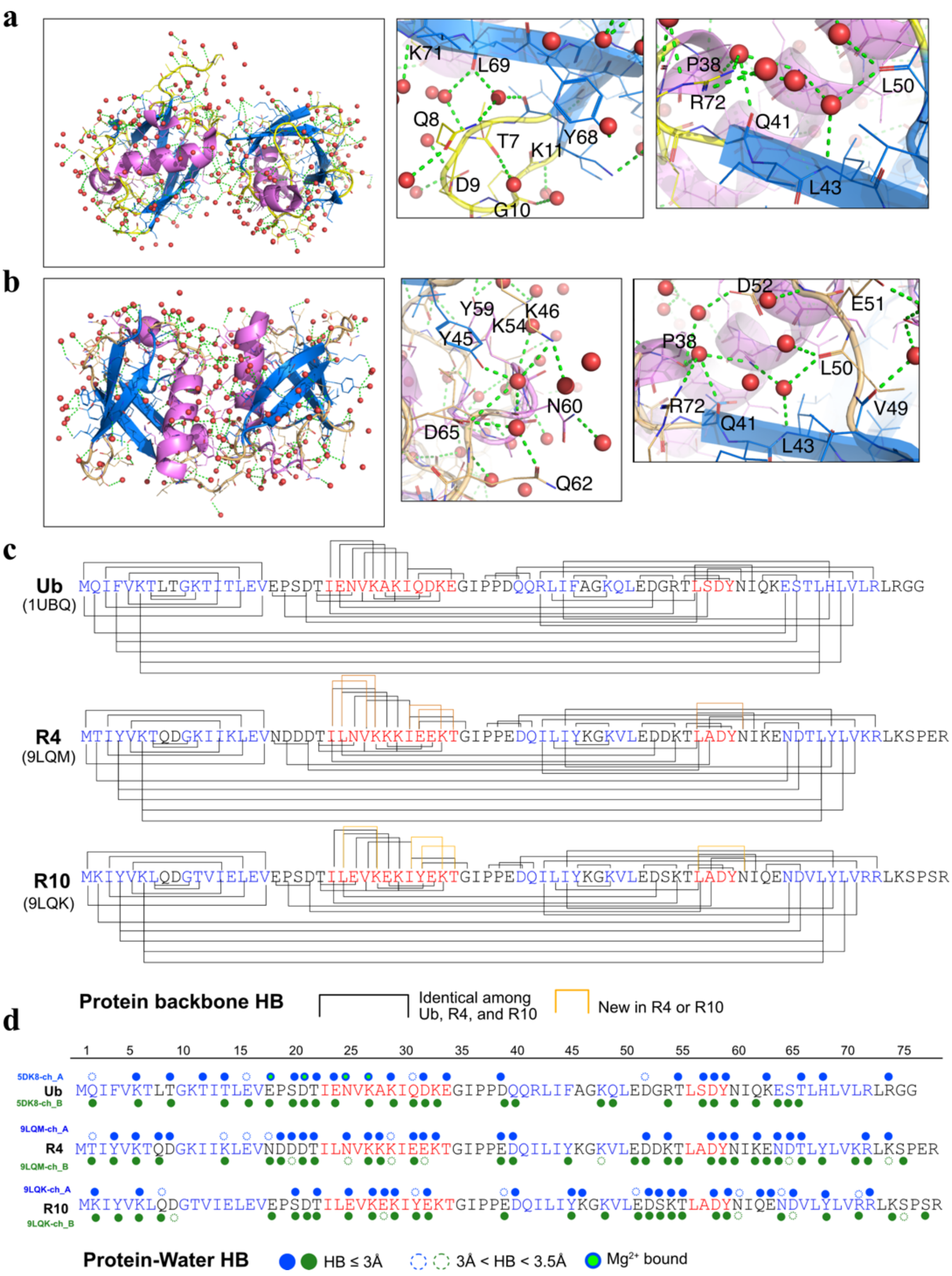

a
K71
L69
Q8
T7
K11
Y68
D9
G10
P38
R72
Q41
L50
L43
b
Y59
K46
K54
Y45
N60
D65
Q62
D52
E51
P38
L50
R72
Q41
V49
L43
c
Ub
(1UBQ)
MQIFVKTLTGKTITLEVEPSDTIENVKAKIQDKEGIPPDQQRLIFAGKQLEDGRTLSDYNIQKESTLHLVLRLRGG
R4
(9LQM)
MTIYVKTQDGKIIKLEVNDDDTILNVKKKIEEKTGIPPEDQILIYKGKVLEDDKTLADYNIKENDTLYLVKRLKSPER
R10
(9LQK)
MKIYVKLQDGTVIELEVEPSDTILEVKEKIYEKTGIPPEDQILIYKGKVLEDSKTLADYNIQENDVLYLVRRLKSPSR
Protein backbone HB
Identical among Ub, R4, and R10
New in R4 or R10
d
1 5 10 15 20 25 30 35 40 45 50 55 60 65 70 75
5DK8-ch_A
Ub
5DK8-ch_B
9LQM-ch_A
R4
9LQM-ch_B
9LQK-ch_A
R10
9LQK-ch_B
Protein-Water HB
HB ≤ 3Å
3Å < HB < 3.5Å
Mg²⁺ bound

Figure S2. Water-linked hydrogen bonding networks in Ub, R4, and R10.
The crystal structures of R4 (panel **a**) and R10 (panel **b**) reveal water molecules depicted as red dots. Hydrogen bonds are represented by green dashed lines, and selected residues engaging in solvent hydrogen bonding are presented. The intramolecular hydrogen bonding networks of Ub, R4, and R10 are linked between residues, shown by black lines (panel **c**). Compared to Ub, R4 and R10 exhibit a few new hydrogen bonds, depicted by yellow lines. Residues are colored based on their secondary structure, with blue representing β-strands and red indicating α-helices. In the high-resolution crystal structures of Ub, R4, and R10 (panel **d**), water molecules are shown as blue or green dots, representing the protein-solvent hydrogen bonds for chain A or B, respectively. Weak hydrogen bonds are denoted by open dashed circles. The crystal structure of Ub chain A also has a few interactions with Mg^{2+} and water, as indicated by green-filled circles.

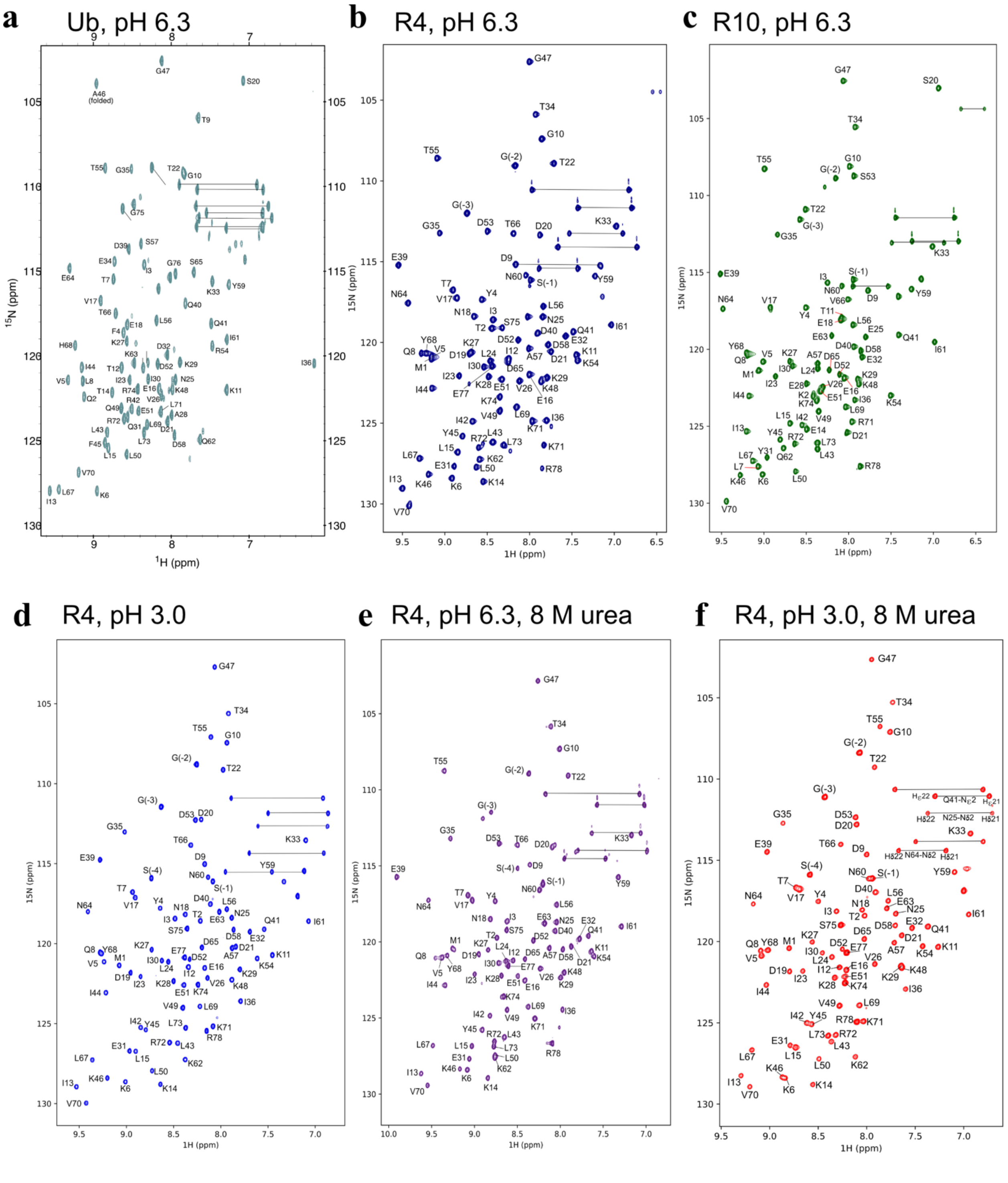

a
Ub, pH 6.3
^{15}N (ppm)
^{1}H (ppm)
b
R4, pH 6.3
15N (ppm)
1H (ppm)
c
R10, pH 6.3
15N (ppm)
1H (ppm)
d
R4, pH 3.0
15N (ppm)
1H (ppm)
e
R4, pH 6.3, 8 M urea
15N (ppm)
1H (ppm)
f
R4, pH 3.0, 8 M urea
15N (ppm)
1H (ppm)

Figure S3. ^{15}N-HSQC spectral assignment of Ub, R4, R10, and related conditions.
A total of six ^{15}N-HSQC spectra were assigned. Panels **(a)**, **(b)**, and **(c)** present the assignments for Ub, R4, and R10, respectively, all at pH 6.3. Assignments for R4 at pH 3.0, pH 6.3 with 8 M urea, and pH 3.0 with 8 M urea are provided in the bottom three panels (**d**, **e**, and **f**). The sidechain NH_2 of the asparagine and glutamine residues are connected by horizontal lines in the spectra.

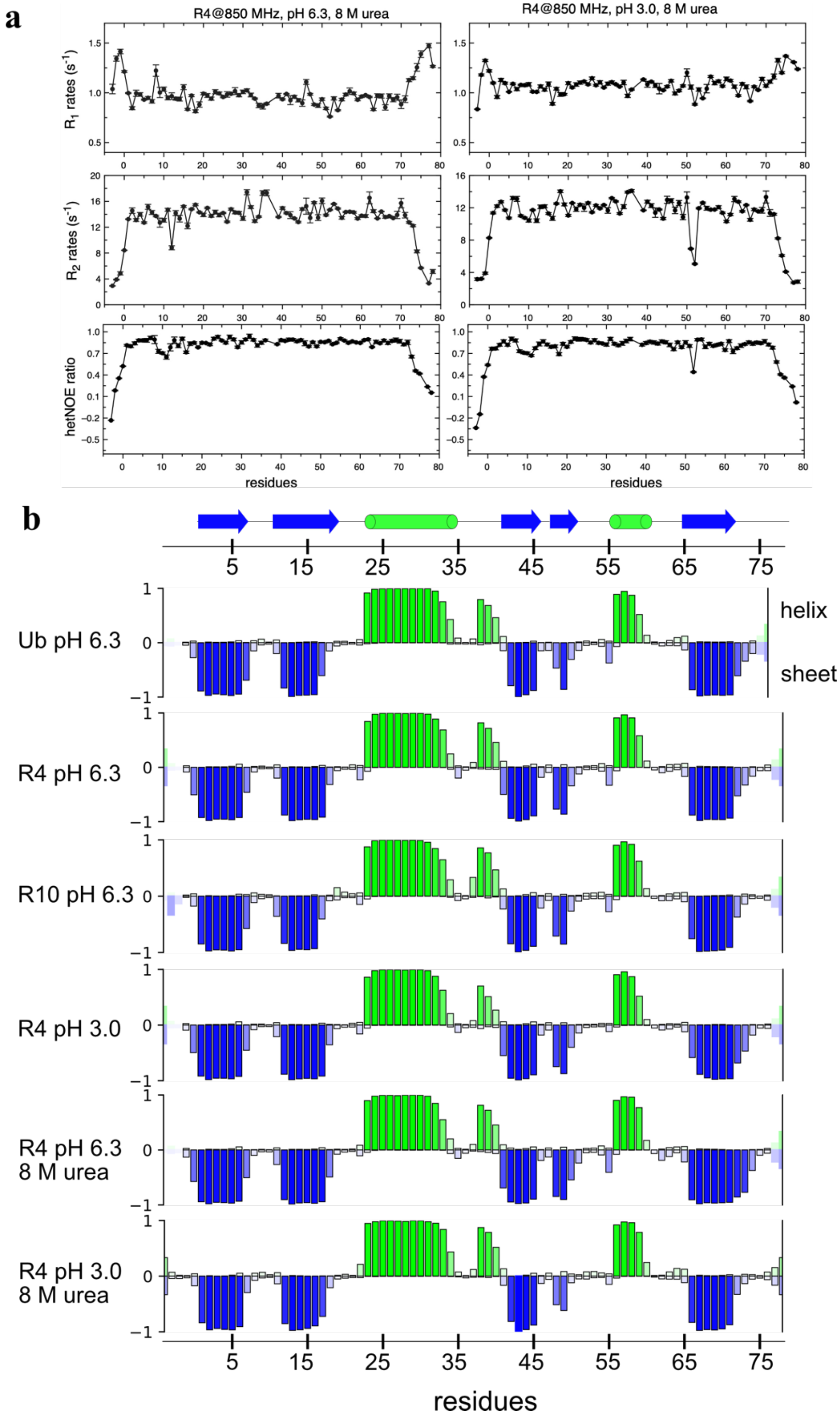
a
R4@850 MHz, pH 6.3, 8 M urea
R4@850 MHz, pH 3.0, 8 M urea
R1 rates (s-1)
R2 rates (s-1)
hetNOE ratio
residues
residues
b
Ub pH 6.3
R4 pH 6.3
R10 pH 6.3
R4 pH 3.0
R4 pH 6.3
8 M urea
R4 pH 3.0
8 M urea
helix
sheet
residues

Figure S4. NMR analysis of Ub, R4, and R10 under varied conditions.
(a). The ^{15}N relaxation parameters, including R_1, R_2, and hetNOE, of R4 under two different stress conditions. **(b).** The secondary structural propensities and regions of Ub, R4, and R10 were predicted using TALOS-N under varied conditions. Blue and green bars depict the β-strand and α-helix, respectively.

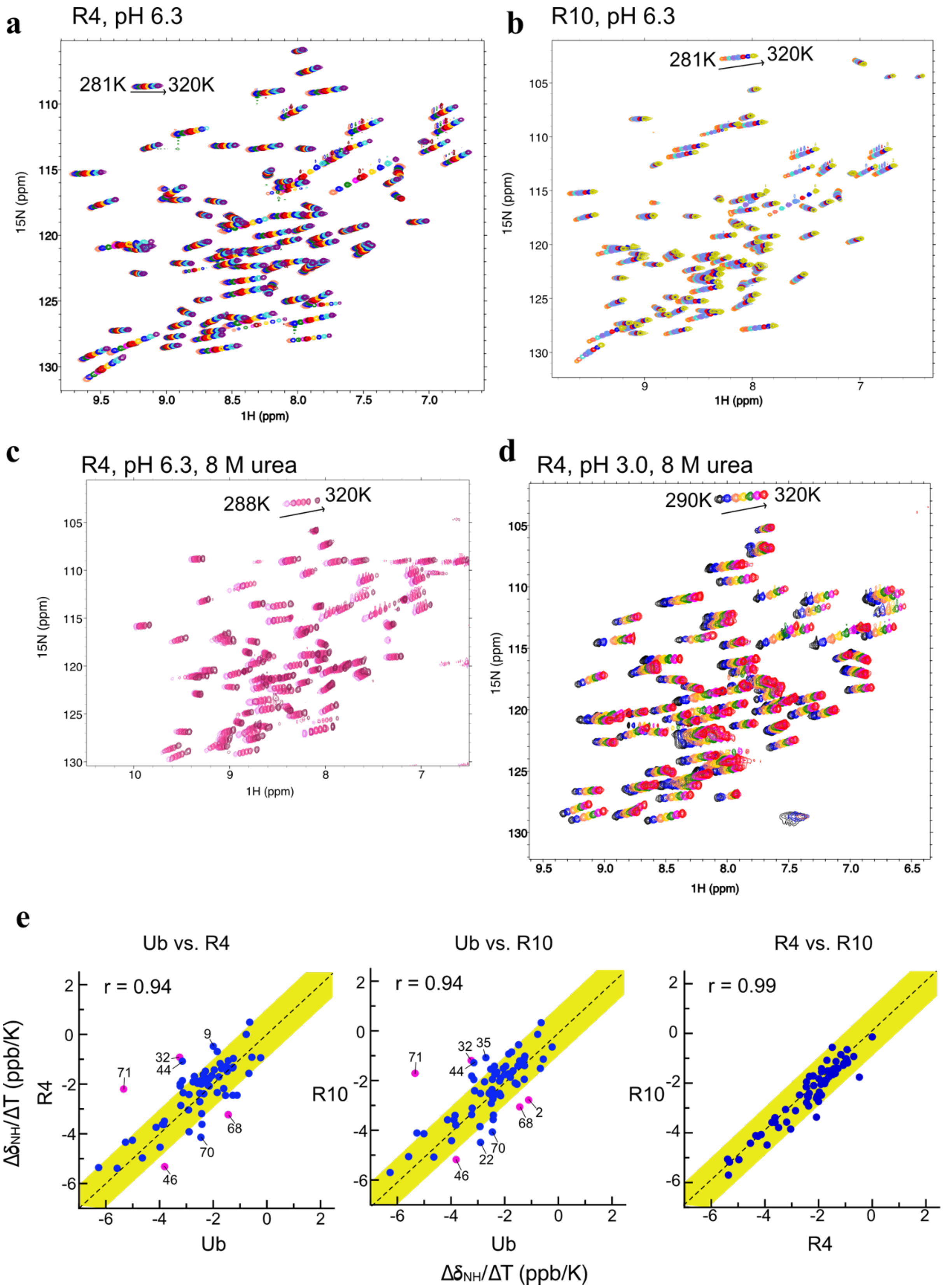
a
R4, pH 6.3
281K
320K
15N (ppm)
1H (ppm)
b
R10, pH 6.3
281K
320K
15N (ppm)
1H (ppm)
c
R4, pH 6.3, 8 M urea
288K
320K
15N (ppm)
1H (ppm)
d
R4, pH 3.0, 8 M urea
290K
320K
15N (ppm)
1H (ppm)
e
Ub vs. R4
r = 0.94
Ub
R4
Ub vs. R10
r = 0.94
Ub
R10
R4 vs. R10
r = 0.99
R4
R10
ΔδNH/ΔT (ppb/K)
ΔδNH/ΔT (ppb/K)

Figure S5. Temperature dependence of R4 and R10 backbone amides
^{15}N-HSQC spectra were collected for R4 and R10 proteins under four different conditions, spanning a temperature range of 281-320K. This included R4 at pH 6.3 (**a**), R10 at pH 6.3 (**b**), R4 at pH 6.3 with 8 M urea (**c**), and R4 at pH 3.0 with 8 M urea (**d**). The spectra exhibit a color gradient, with arrows indicating the transition from low to high temperatures. (**e**). Correlation plots of residue-specific temperature coefficients ($\Delta\delta NH/\Delta T$) for Ub, R4, and R10 at pH 6.3. Pearson correlation coefficients (r) are indicated in each subplot. The yellow band denotes values within ±1 ppb/K deviation from the diagonal line. Pink dots in the Ub–R4 and Ub–R10 comparisons highlight residues with notable differences; for example, L71K in R4 and L71R in R10 show the largest elevations, approximately +3 ppb/K.

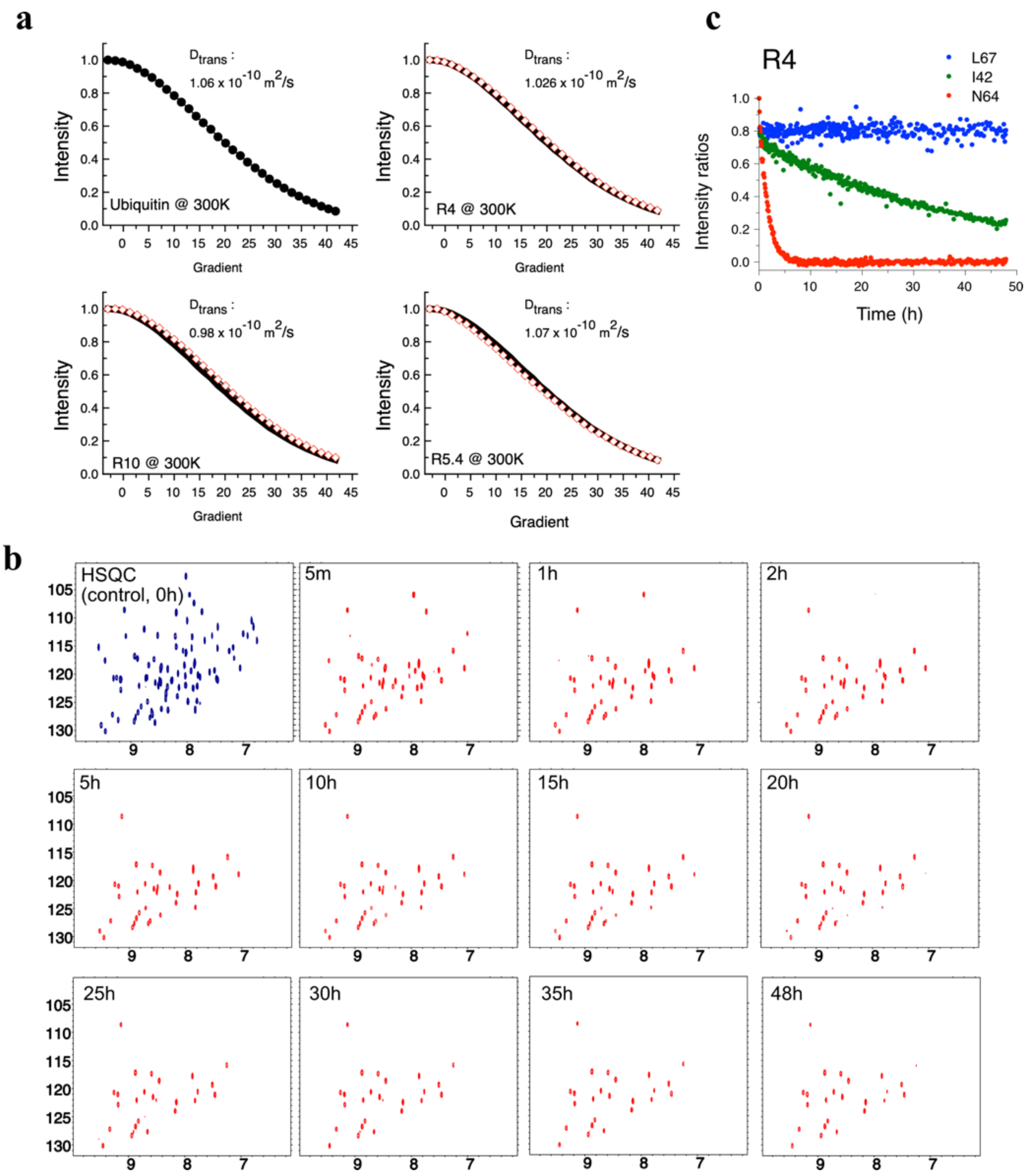
a
Ubiquitin @ 300K
R4 @ 300K
R10 @ 300K
R5.4 @ 300K
Intensity
Gradient
1.06 x 10-10 m2/s
1.026 x 10-10 m2/s
0.98 x 10-10 m2/s
1.07 x 10-10 m2/s
b
HSQC (control, 0h)
5m
1h
2h
5h
10h
15h
20h
25h
30h
35h
48h
c
R4
L67
I42
N64
Intensity ratios
Time (h)

Figure S6. NMR translational diffusion coefficients and HDX data
(a). The translational diffusion coefficients (D_{trans}) of Ub, R4, R10, and phage-displayed R5.4 were measured using 2D DOSY experiments. The integrated volumes of selected peaks were plotted and fitted across a range of gradient strengths, as described in the Methods. The measured D_{trans} values of Ub and R5.4 are identical, whereas R4 and R10 exhibit slight decreased values, indicative of the same monomeric state and a subtly altered protein hydrodynamic radius. **(b).** Examples of twelve ^{15}N-HSQC spectra of R4 before and after hydrogen-deuterium exchange over 48 hours. The spectra are presented at a constant threshold, demonstrating the decreasing peak intensity over time. **(c).** Selected residues N64 and I42 represent fast and slow intensity changes, respectively. The peak intensity of L67 remained at ~80% that of the control peak since the first HDX experiment, indicating minimal exchange of the amide proton.

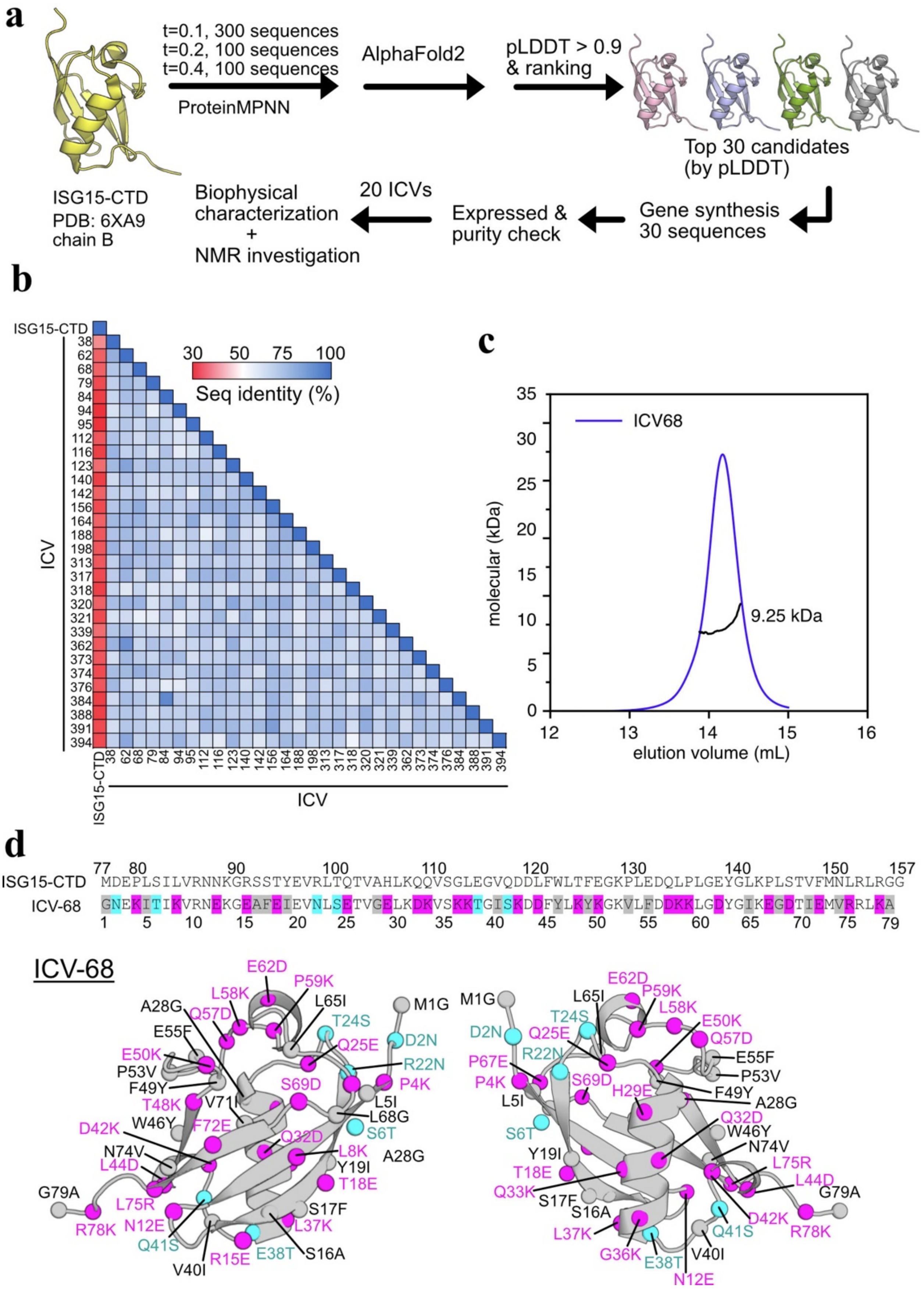

a
t=0.1, 300 sequences
t=0.2, 100 sequences
t=0.4, 100 sequences
ProteinMPNN
AlphaFold2
pLDDT > 0.9
& ranking
Top 30 candidates
(by pLDDT)
ISG15-CTD
PDB: 6XA9
chain B
Biophysical characterization
+
NMR investigation
20 ICVs
Expressed & purity check
Gene synthesis
30 sequences
b
ISG15-CTD
30 50 75 100
Seq identity (%)
ICV
c
ICV68
9.25 kDa
molecular (kDa)
elution volume (mL)
d
ISG15-CTD MDEPLSILVRNNKGRSSTYEVRLTQTVAHLKQQVSGLEGVQDDLFWLTFEGKPLEDQLPLGEYGLKPLSTVFMNLRLRGG
ICV-68 GNEKITIKVRNEKGEAFEIEVNLSETVGELKDKVSKKTGISKDDFYLKYKGKVLFDDKKLGDYGIKEGDTIEMVRRLKA
ICV-68

Figure S7. Selection and characterization of ProteinMPNN-designed ISG15-CTD variants (ICVs).
(**a**) Workflow for the generation and selection of ISG15-CTD variants. The crystal structure of ISG15-CTD (PDB ID: 6XA9) was used as the structural template for ProteinMPNN sequence design. Following metric-based filtering, 30 ICVs were selected for gene synthesis and experimental characterization (sequences, see table S3). (**b**) Sequence identities of the 30 selected ICVs range from 39–47% relative to the ISG15-CTD template, while the identities among ICVs are 60–90%. (**c**) Analytical characterization of ICV-68 confirming a monomeric species in solution. **(d**) Mapped substitutions from ISG15-CTD to ICV-68 shown in both aligned sequences and AlphaFold predicted structures. The substitutions are colored in pink, cyan, and gray balls represent to charged, polar, and nonpolar residues, respectively.

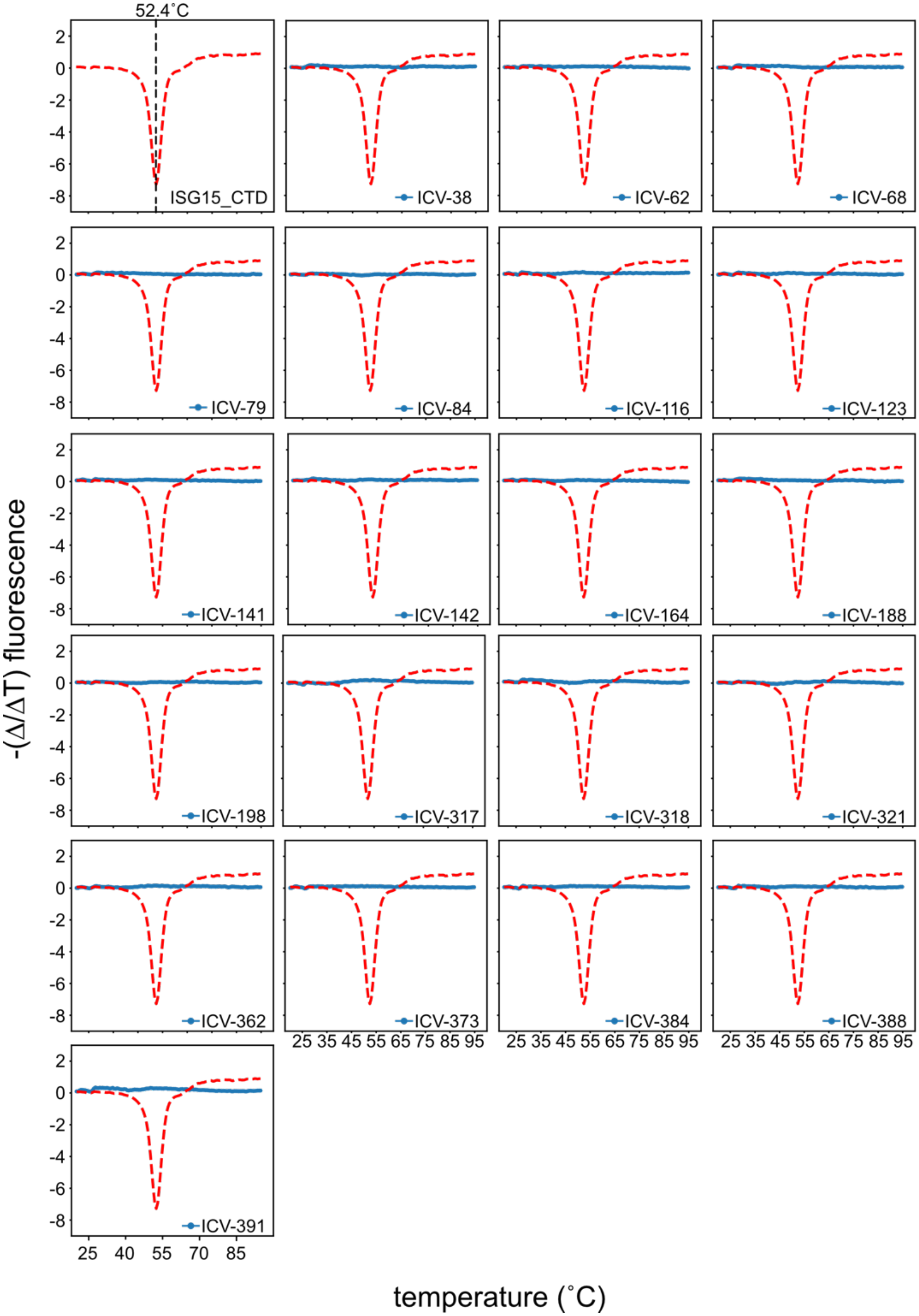
52.4˚C
ISG15_CTD
ICV-38
ICV-62
ICV-68
ICV-79
ICV-84
ICV-116
ICV-123
ICV-141
ICV-142
ICV-164
ICV-188
ICV-198
ICV-317
ICV-318
ICV-321
ICV-362
ICV-373
ICV-384
ICV-388
ICV-391
-(Δ/ΔT) fluorescence
temperature (˚C)
2
0
-2
-4
-6
-8
25 35 45 55 65 75 85 95
25 40 55 70 85

Figure S8. Thermal stability of ProteinMPNN-designed ISG15 CTD variants.
Thermal stability of 20 ICVs determined by differential scanning fluorimetry (DSF). The T_m of ISG15-CTD was calculated to be 52.4 °C, consistent with its thermal unfolding transition. In contrast, all 20 expressed ICVs exhibited remarkable thermal stability, showing no apparent denaturation even when heated up to 95 °C. In each plot, the thermal shift curve of ISG15-CTD, shown in red, is overlaid with that of the corresponding ICV for direct comparison. Notably, all 20 ICVs displayed minimal fluorescence change across the entire temperature range from 25 °C to 95 °C (blue dotted lines), suggesting that their apparent T_m exceed 95 °C, indicative of exceptional thermostability conferred by ProteinMPNN design

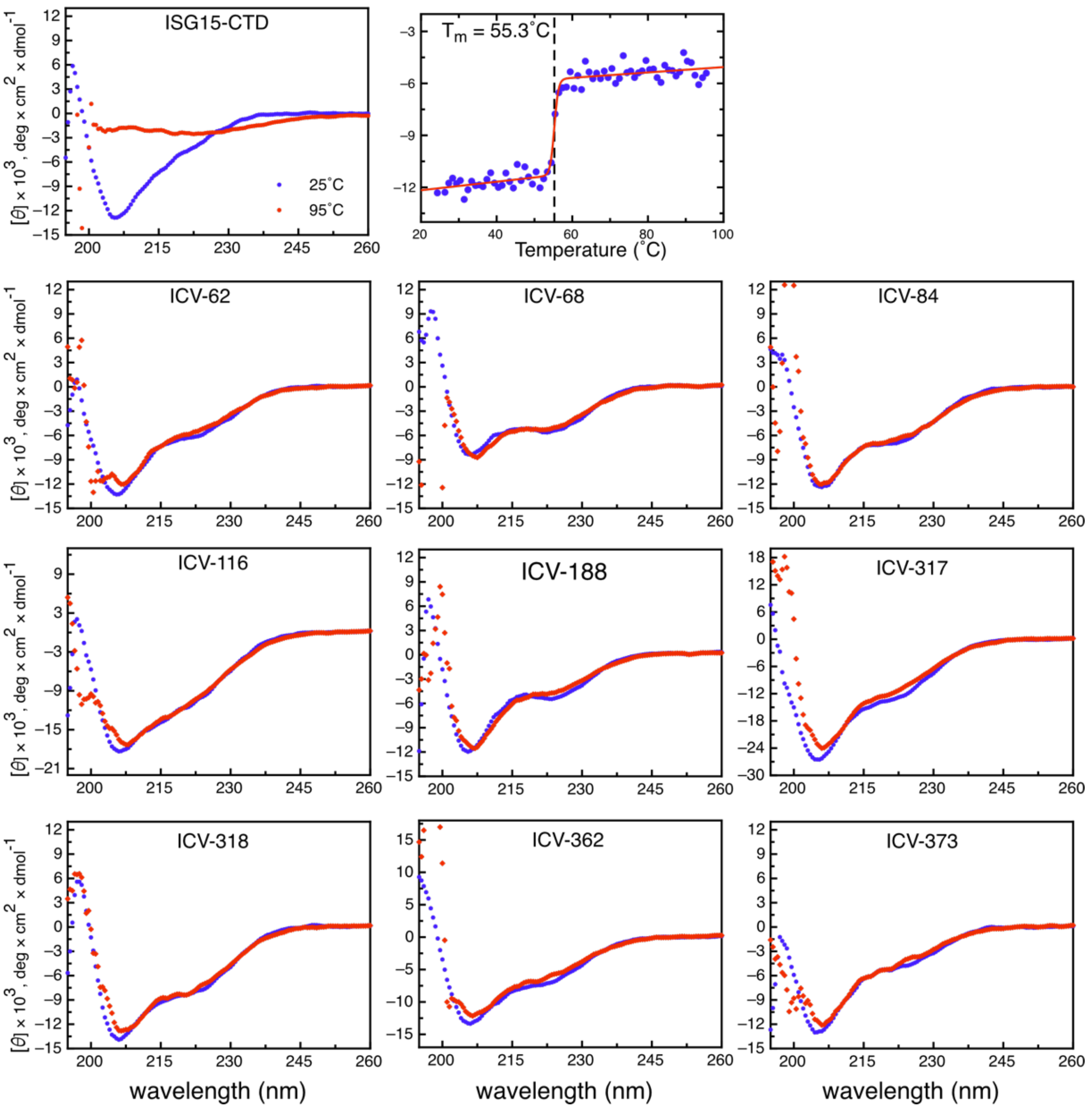

Figure S9. Thermal stability of ProteinMPNN-designed ISG15 CTD variants.
ISG15-CTD and six selected ICVs were further analyzed by circular dichroism (CD) spectroscopy. Upon heating from 25 to 95 °C, ISG15-CTD underwent denaturation, with a melting temperature of 55.3 °C—consistent with the Tm values obtained from the thermal shift assay (Figure S8) and DSC measurements (Figure 1a). In contrast, the CD spectra of the six ICVs at 25 °C and 95 °C were nearly identical, highlighting the exceptional heat resistance of the ProteinMPNN-designed ICVs.

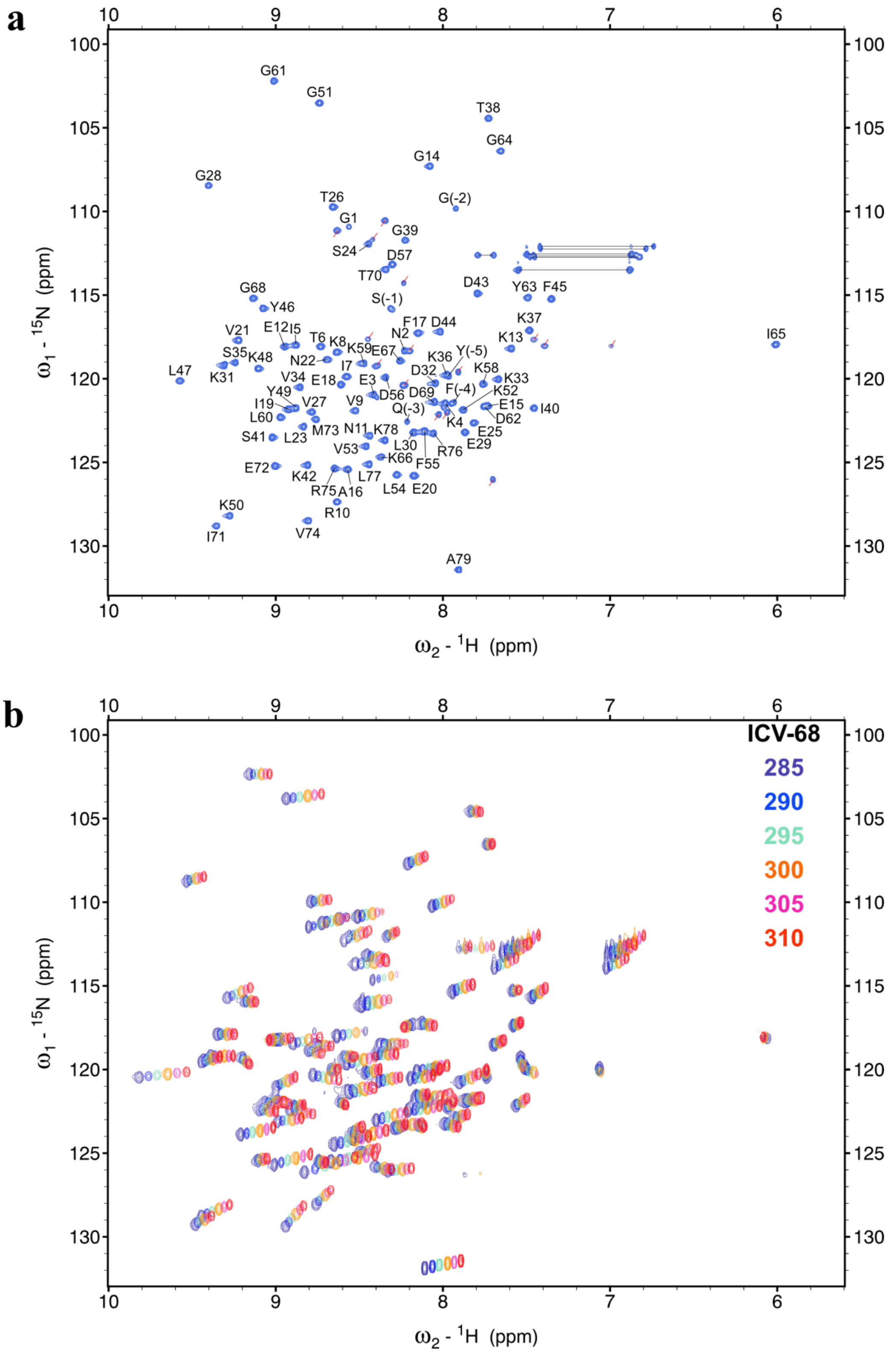

Figure S10. NMR HSQC assignment and temperature-dependent changes of ICV-68.
(**a**) The HSQC spectrum of ICV-68 recorded at 300 K on an 850 MHz spectrometer was fully assigned. (b) To determine the residual temperature coefficients ($\Delta\delta_{NH}/\Delta T$), six HSQC spectra of ICV-68 were collected over a temperature range from 285 K to 310 K in 5 K increments.

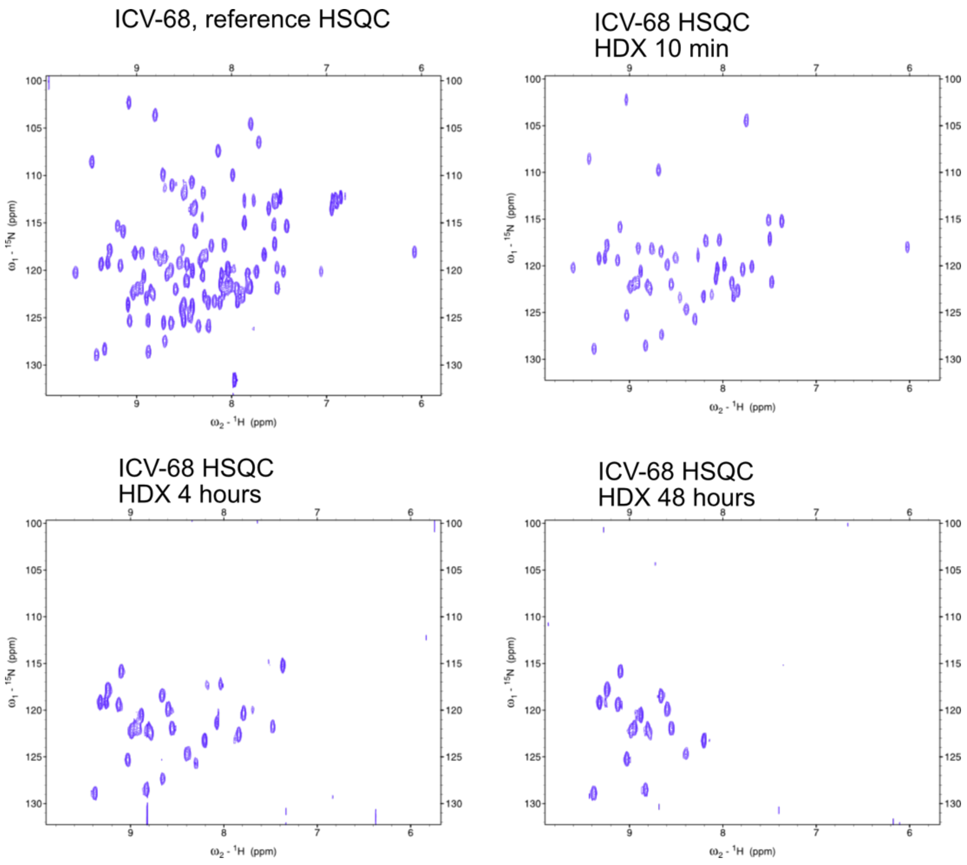

Figure S11. Hydrogen-deuterium exchange spectra of ICV-68.
The HDX-NMR experiments of ICV-68 were performed at 300 K on a 600 MHz spectrometer to monitor time-dependent changes. Ten minutes after transferring the sample into D_2O buffer, the intensities of most crosspeaks decreased but remained clearly detectable. The subsequent exchange rates were notably slow, as only minor changes were observed between the 4-hour and 48-hour HDX spectra. More than 15 crosspeaks remained clearly visible even after 48 hours of HDX, indicating high structural stability of ICV-68.

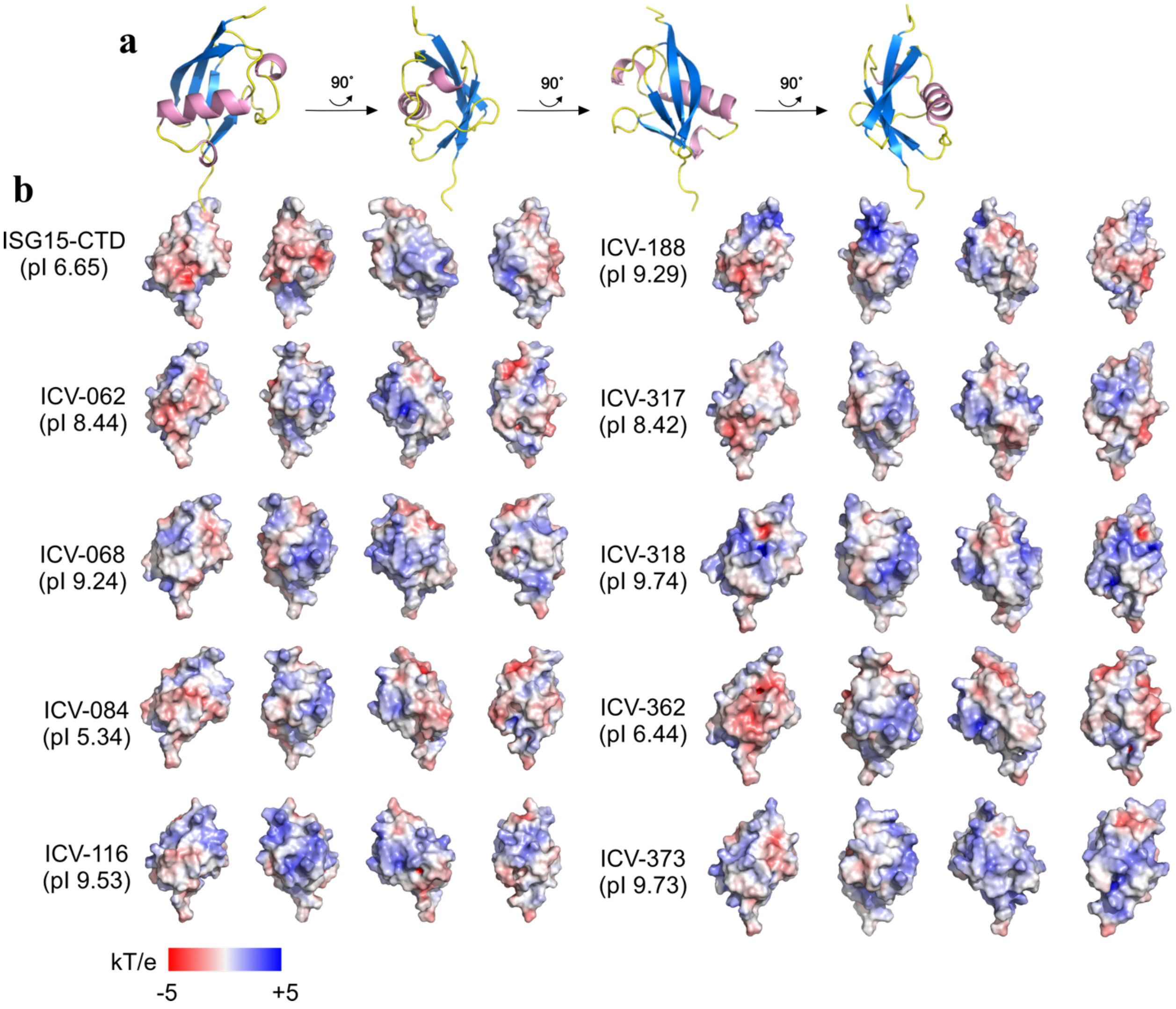

Figure S12. Electrostatic states of ICV proteins
(**a**) Four representative orientations of the ISG15-CTD structure are shown to illustrate its overall topology and domain organization. (**b**) The electrostatic surface potentials of ISG15-CTD and nine selected ICVs are presented for comparison, with negatively charged regions shown in red and positively charged regions in blue. These maps reveal that, while the ProteinMPNN-designed ICVs preserve the overall structural framework of ISG15-CTD, they display distinct surface charge distributions that may influence their solubility, interaction properties, and thermodynamic stability.

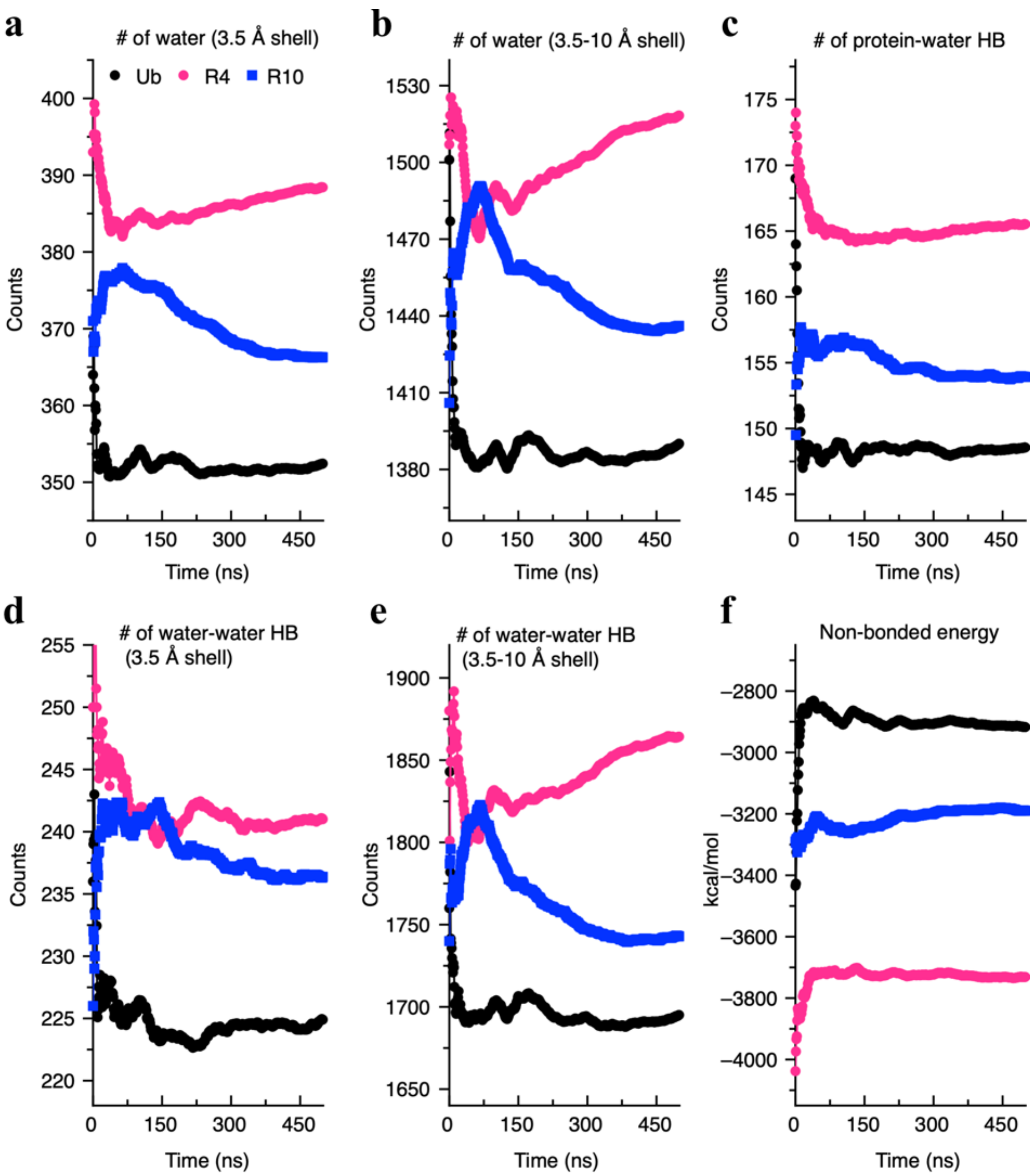

Figure S13. Analysis of MD-simulated hydration of assessed proteins
The MD simulation data for Ub, R4, and R10 are shown as black, pink, and blue dots, respectively. Counts of water molecules, hydrogen bonds, and energy were plotted using cumulative averages over time. In the primary (0 – 3.5 Å, panel **a**) and secondary shells (3.5 – 10 Å, panel **b**), Ub has significantly fewer water molecules than R4 and R10. Similarly, R4 and R10 exhibit more protein-water (panel **c**) and water-water hydrogen bonds (panels **d**, **e**) compared to Ub. Consequently, R4 is more stable than R10 and Ub, with Ub being relatively less stable by approximately 700 kcal/ml (panel **f**).

Table S1. Data collection and structural quality of crystal structures R4 and R10

**Data collection**	R4	R10
**PDB accession code**	9LQM	9LQK
Source	NSRRC TPS07A	NSRRC TPS07A
Wavelength (Å)	1.0	1.0
Space group	$P3_221$	*I222*
Cell dimensions		
a, b, c (Å)	50.84, 50.84, 102.67	57.56, 62.55, 89.76
α, β, γ (°)	90.00, 90.00, 120.00	90.00. 90.00, 90.00
Resolution (Å)	25.42-1.34 (1.39-1.34)	30.00-1.55 (1.61-1.55)
Completeness (%)	99.6 (77)	97.1 (90.8)
Total reflections	5812808	16747556
Unique reflections	31670 (3126)	23160 (2137)
Wilson B-factor	13.45	22.63
Multiplicity	17.7 (17.0)	4.9 (4.2)
R_{merge} (%)	7.7 (71.5)	6.0 (41.6)
< I >/σ(I)	37.37 (4.01)	23.29 (2.63)
**Refinement and validation**		
Resolution (Å)	25.42 - 1.39 (1.43 - 1.39)	25.658 - 1.550 (1.606 - 1.550)
Reflections (work/free)	34192 (3027)	23078 (2061)
R_{work} (%)	16.36 (18.65)	19.14 (30.33)
R_{free} (%)	19.50 (23.52)	22.87 (34.95)
Number of atoms	1540	1456
Protein	1278	1275
Ligand	25	6
Water	237	175
Average *B*-factors (Å^2)	22.0	32.93
Protein	19.61	31.52
Ligand	51.83	38.12
Water	37.58	42.65
RMSD		
Bond lengths (Å)	0.0090	0.0065
Bond angles (°)	1.16	0.90
MolProbity		
Favored (%)	98.73	100
Allowed (%)	1.27	0.00
Outliers (%)	0.00	0.00
Clash score	0.76	2.29
MolProbity score	1.05	1.01

Note: values in parentheses are for the highest-resolution shell.

Table S2. NMR assignment and structure determination of R4, R10, and Ub

Proteins	R4	R10	R4	R4	Ub	R4
BMRB deposition number	36739	36738	36737	36736		
PDB accession code	9M9H	9M9G	9M8X	9M8W		
**Experimental conditions**	pH 6.3	pH 6.3	pH 6.3, 8 M urea	pH 3.0, 8 M urea	pH 6.3	pH 3.0
**NMR assignment statistics**						
^{1}H (%)	98.6	98.1	92.6	91.2	97.2	91.9
^{13}C (%)	89.1	88.0	85.0	87.0	89.4	90.3
^{15}N (%)	81.9	82.3	75.2[(a)]	72.4[(a)]	81.3	75.2[(a)]
All ^{1}H,^{13}C, ^{15}N atoms (%)	93.7	93.1	88.3	88.0	92.9	88.3
**Structure calculation**						
Total residue number [(b)]	83	83	83	83		
Total number of NOE restraints	1386	1562	1635	1875		
Intra-residues	499	430	611	736		
Sequential $\| i\text{-}j \|=1$	333	402	307	351		
Medium range $1<\| i\text{-}j \| <5$	199	253	260	269		
Long range $\| i\text{-}j \| \geq 5$	355	477	457	519		
Total hydrogen bond restraints	27	35	71	55		
Sequential $\| i\text{-}j \|=1$	0	0	0	0		
Medium range $1<\| i\text{-}j \| <5$	14	16	39	35		
Long range $\| i\text{-}j \| \geq 5$	13	19	32	20		
Dihedral angle restraints						
Phi/Psi (˚)	73/73	70/70	72/72	70/70		
**Ensemble analysis (20 structures)**						
RMSD from ideal geometry						
Bond length (Å)	0.003	0.003	0.004	0.005		
Bond angle (˚)	0.6	0.5	0.6	0.7		
Improper contacts	0	0	0	0		
RMSD from the mean structure (Å)						
Backbone (structured) [(c)]	0.3	0.3	0.6	0.5		
Backbone (all)	0.9	1.5	1.2	1.0		
Heavy atoms (structured)	0.7	0.7	1.1	1.1		
Heavy atoms (all)	1.3	1.7	1.6	1.6		
Ramachandran statistics (%)						
Most favored regions	95.6	97.3	94.9	92.3		
Additional allowed region	2.3	2.1	3.5	6.0		
Disfavored region	2.0	0.6	1.7	1.6		
Global quality scores						
Molprobity Clashscore	6	4	10	14		

Notes:

(a) Nitrogen atoms in Asn, Gln sidechains were neglected for spectral assignment

(b) The cloning artifact GSGGS residues as the N-terminus are included.

(c) Residues 1-72 are defined as structured region

Table S3. Protein sequences of selected ISG15-CTD variants

Protein	Sequence
ISG15-CTD	MDEPLSILVRNNKGRSSTYEVRLTQTVAHLKQQVSGLEGVQDDLFWLTFEGKPLEDQLPLGEYGLKPLSTVFMNLRLRGG
ICV-38	GDEAITIKVQNEKGESFSFTVKLSDTVAELKKQVSEKTGINEDDFYLTYKGKVLFDDKKLGEYGIKENDTIKMVRRLKA
ICV-62	GNEKITIKVTNEKGESFEFTVNLSETVSELKKKVSEKTGIDESDFYLTYKGKVLEDSKALGTYGIKENDTIKMVRRLKA
ICV-68	GNEKITIKVRNEKGEAFEIEVNLSETVGELKDKVSKKTGISKDDFYLKYKGKVLFDDKKLGDYGIKEGDTIEMVRRLKA
ICV-79	GNADITIKVRNEEGKEIEITVNLSDTVAELRKKVSEKTGIDEEDFYLKYKGKVLEDSKALGEYGIKEGDTIEMVRRLKA
ICV-84	GNEKITIKVENEQGEAIEIEVNLSEKVSVLKEKISEKTGINKEDFYLTYKGKVLEDEKKLGEYGIKENDVIKMVRRLKA
ICV-94	SNKKITIKVQNEKGEEKEFEVNLDETVGELKEKVSKEFGIDKNDFYLTYKGKVLKDEKKLKDYGIKENDVIKMVRRLKA
ICV-95	GMHEITILVKDENGNAFEFKVNLSDTVGELKKKVSEKTGINEEDFYLTYKGKVLEDEKALGSYGIKEGDVIEMVRRLKA
ICV-112	GNADITIKVEDENGKSYSFTVNLNDTVGKLKDQVSAKTGIDKEDFYLTYKGKVLEDDKKLGEYGIKENDTIKMVRRLKA
ICV-116	GNEAITIKVQDEEGKAYSFTVKLSEKVSTLKEKVSEKTGINKDDFYLTYKGKVLFDDKKLGEYGIKEGDTIKMIRRLKA
ICV-123	SNEEITIKVENEKGESIEFKVNLDDTVGELKKKVSEKTGIDESDFYLTYKGKVLEDSKKLGEYGIKEGDTIKMVRRLKA
ICV-140	GNEDITILVKDENGKAHSFTVNLDETVSELKDKVSEKTGIDKDDFYLTYKGKVLEDSKKLKDYGIKENDVINMVRRLKA
ICV-142	SNEKITINVRDENGKSIKFEVNLDDTVSELKDQVSKKTGIDKSDFYLTYKGKVLFDDKKLKEYGIKENDTINMVRRLKA
ICV-156	GMEKITIKVEDENGKAYEFEVNLSETVGELKDKVSEKTGIDKSDFYLTYKGKVLFDDKKLGEYGIKEGDTIKMVRRLKA
ICV-164	GMEEITILVRNEKGEAMEFKVNLSETVGELKKKVSEKTGIDEEDFYLTYKGKVLFDDKKLGEYGIKEGDVIKMVRRLKA
ICV-188	GMHSITIKVRNEKGEEHEFKVKLSETVAELKKKVSEKFGIDESDFYLTYKGKVLFDSEKLGSYGIKEGDTINMVRRLKA
ICV-198	GDEEITIKVQNEEGKSIEITVKLSETVGELKDKVSEKTGINKEDFYLTYKGKVLFDEKKLGDYGIKEGDTIKMVRRLKA
ICV-313	GNEDITINVTNEQGESFKITVNLSETVGVLKDKVSEKTGIDKEDFYLTYKGKVLEDGKALGEYGIQEGDTIKMVRRLKA
ICV-317	GNAEITIKVEDENGKAYSFKVNLSETVASLKKKVSEKTGIDESDFYLTYKGKVLEDTKALGEYGIKEGDTIKMVRRLKA
ICV-318	GDAEITILVKNEEGKSISIKVKLSETVGKLKELVSKKTGIDKNDFYLTYKGKVLFDDKKLGDYGIKENDVINMVRKLKA
ICV-320	GNEEITIYVENEKGESFKFKVNLDETVGELKDKVSKKTGIDKNDFYLTYKGKVLKDDKKLGEYGIKENDTIKMVRRLKA
ICV-321	GNEEITILVEDYEGKAHSFTVKLTEKVSTLKDKVSEKFGIDKEDFYLTFKGKVLFDEKALGEYGIKENDVIKMVRRLKA
ICV-339	GNERITILVEDEEGKSFSFEVRLSETVGWLKKQVSEKTGIDESDFYLTYKGKVLEDDKRLGDYGIKEGDVIKMVRRLKA
ICV-362	GNEKITIKVTNEKGESIDFTVNLSDTVSELKKLISEKTGIDENDFYLTYKGKVLEDGKALGTYGIKENDTIKMVRRLKA
ICV-373	GNKKITIKVKNAEGKSFEFEVNLDEKVSELKEKVSKKTGIDKNDFYLTYKGKVLEDEKRLGEYGIKENDTINMVRRLKA
ICV-374	GNADITIKVENEKGEAIEFTVNLSDTVGELKDKVSEKTGINKDDFYLTYKGKVLLDDKALGDYGIKAGDTIKMVRRLKA
ICV-376	GDEEITIYVEDEEGKKHKFTVKLSDTVGELKKKVSKKTGIDENDFYLTYKGKVLFDDKKLGEYGIKENSTIKMIRRLKA
ICV-384	GNEKITIKVENAQGKAIEFEVNLSEKVSELKEKISEKTGISKEDFYLTYKGKVLEDEKKLGEYGIKENDVIKMVRRLKA
ICV-388	GMEDITIKVEDEEGKSFSFKVNLSETVGELKEKVSKKTGIAKEDFYLTYKGKVLKDEEKLGKYGIKENDTIKMVRRLKA
ICV-391	GNAKITIFVEDEEGKSYKFEVNLDETVGELKDKVSEKTGIDKSDFYLTYKGKVLKDEKKLGEYGIKEGDTIKMIRRLKA
ICV-394	SNEKITIKVQNEKGEEHEFEVNLSETVGELKKKVSEKFGIDEEDFYLTYKGKVLEDSKALGNYGIQENDVIKMVKRLKA
